# *In Silico*, *In Vitro*, and *In Vivo* Evaluation of Precipitation Inhibitors in Supersaturated Lipid-Based Formulations of Venetoclax

**DOI:** 10.1021/acs.molpharmaceut.0c00645

**Published:** 2021-04-23

**Authors:** Niklas J. Koehl, Laura J. Henze, Harriet Bennett-Lenane, Waleed Faisal, Daniel J. Price, René Holm, Martin Kuentz, Brendan T. Griffin

**Affiliations:** † School of Pharmacy, University College Cork, College Road, T12 YN60 Cork, Ireland; ‡ Drug Product Development, Janssen Research and Development, Johnson & Johnson, Turnhoutseweg 30, 2340 Beerse, Belgium; § Analytical Development, Janssen Research and Development, Johnson & Johnson, Turnhoutseweg 30, 2340 Beerse, Belgium; ∥ Merck KGaA, Frankfurter Str. 250, 64293 Darmstadt, Germany; ⊥ Institution of Pharmaceutical Technology, Goethe University Frankfurt, Max-von-Laue-Strasse 9, 60439 Frankfurt am Main, Germany; # Department of Science and Environment, Roskilde University, 4000 Roskilde, Denmark; ¶ Department of Physics, Chemistry and Pharmacy, University of Southern Denmark, Campusvej 55, 5230 Odense, Denmark; ∇ Institute of Pharma Technology, University of Applied Sciences and Arts Northwestern Switzerland, Hofackerstrasse 30, 4132 Muttenz, Switzerland; ○ Faculty of Pharmacy, Minia University, Minia, Egypt

**Keywords:** precipitation inhibitor, lipid based formulation, venetoclax, SEDDS, SNEDDS, SMEDDS, lipid suspension, polymers, super-SNEDDS, supersaturation, super-SMEDDS, supersaturating drug delivery systems

## Abstract

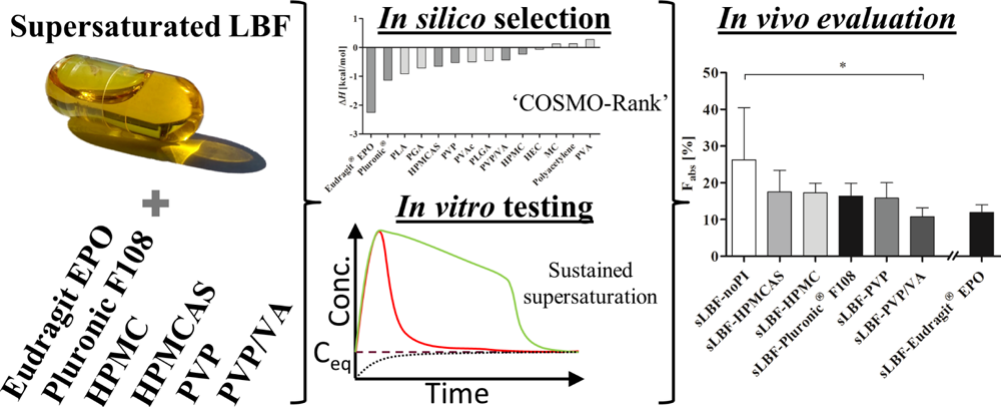

The concept of using precipitation inhibitors (PIs) to sustain supersaturation is well established for amorphous formulations but less in the case of lipid-based formulations (LBF). This study applied a systematic *in silico*–*in vitro*–*in vivo* approach to assess the merits of incorporating PIs in supersaturated LBFs (sLBF) using the model drug venetoclax. sLBFs containing hydroxypropyl methylcellulose (HPMC), hydroxypropyl methylcellulose acetate succinate (HPMCAS), polyvinylpyrrolidone (PVP), PVP-*co*-vinyl acetate (PVP/VA), Pluronic F108, and Eudragit EPO were assessed *in silico* calculating a drug–excipient mixing enthalpy, *in vitro* using a PI solvent shift test, and finally, bioavailability was assessed *in vivo* in landrace pigs. The estimation of pure interaction enthalpies of the drug and the excipient was deemed useful in determining the most promising PIs for venetoclax. The sLBF alone (i.e., no PI present) displayed a high initial drug concentration in the aqueous phase during *in vitro* screening. sLBF with Pluronic F108 displayed the highest venetoclax concentration in the aqueous phase and sLBF with Eudragit EPO the lowest. *In vivo*, the sLBF alone showed the highest bioavailability of 26.3 ± 14.2%. Interestingly, a trend toward a decreasing bioavailability was observed for sLBF containing PIs, with PVP/VA being significantly lower compared to sLBF alone. In conclusion, the ability of a sLBF to generate supersaturated concentrations of venetoclax *in vitro* was translated into increased absorption *in vivo*. While *in silico* and *in vitro* PI screening suggested benefits in terms of prolonged supersaturation, the addition of a PI did not increase *in vivo* bioavailability. The findings of this study are of particular relevance to pre-clinical drug development, where the high *in vivo* exposure of venetoclax was achieved using a sLBF approach, and despite the perceived risk of drug precipitation from a sLBF, including a PI may not be merited in all cases.

## Introduction

Favorable solubility in gastrointestinal fluids and intestinal permeability is a prerequisite for the high oral bioavailability of any drug. However, drug discovery approaches such as high throughput screenings, modifications during lead optimization, as well as the noticeable therapeutic target shift toward intracellular targets deliver more drugs, displaying low aqueous solubility and beyond rule-of-five properties.^1−3^ These drug candidates have sub-optimal biopharmaceutical properties, which typically create a need for bioenabling formulation approaches. The design of such formulations includes strategies to generate and maintain high concentrations or supersaturation in intraluminal fluids. Prominent examples of such supersaturating formulations are amorphous solid dispersions and the use of lipid-based formulations (LBF).^4,5^ In particular, for drugs with a low aqueous solubility, a high lipophilicity and/or bioavailability that is increased by the co-ingestion of fatty meals, LBFs can offer particular formulation advantages.^4−6^ The most convenient and conventional LBFs are lipid solutions, where the drug is dissolved in the lipid vehicle and hence most widely applicable for drugs that show high lipid solubility. Alternatively, for drugs which display low solubility in lipid vehicles, strategies to increase dose loading in the lipid vehicles may be required such as lipid suspensions,^7−9^ supersaturated LBFs (sLBF),^10−13^ lipophilic salts,^14−16^ or lipid–hybrid systems.^17−19^

sLBFs can be beneficial in delivering drug candidates of this type and interest in their application has increased over the past years. The most common method to generate drug supersaturation in lipids is by heating excess drugs in the lipids, followed by cooling,^12^ whereupon the amount of dissolved drug in the lipid excipients may exceed the thermodynamic solubility (at lower temperatures) and the LBF becomes supersaturated. A supersaturated formulation should have at least kinetic stability for practical handling, and sLBFs have successfully been applied to improve the oral bioavailability of a number of poorly water-soluble drugs such as cinnarizine, simvastatin, and halofantrine.^10,11,20−22^ A sLBF formulation approach offers a number of advantages, particularly in the early stages of development. These advantages include the ease of preparation, suitability for the ease of dosing in pre-clinical models, and the ability to prepare prototype formulations on a small scale while keeping the development costs low at times where the attrition rate is usually high. Thomas et al. developed a sLBF of simvastatin at 150% of the saturation solubility and assessed bioavailability in dogs.^10^ The oral bioavailability of the sLBF was 1.8-fold higher when compared to the same dose of a LBF solution at 75% of the saturation solubility.^10^ Recently, our group has reported that a sLBF of venetoclax (containing venetoclax at 350% of the apparent saturation solubility) increased oral bioavailability by 2.1-fold when compared to a control LBF (i.e. lipid suspension) and 3.8-fold when compared to the crude drug powder.^13^ Additionally, the venetoclax study demonstrated during the *in vitro* lipolysis testing of the sLBF that the drug concentration of venetoclax in the aqueous digest phase was in the range of the reported amorphous solubility in the fasted state simulated intestinal fluid (FaSSIF).^13^ With such elevated aqueous phase concentrations achieved by a sLBF, the risk of precipitation is deemed high, and hence, it was hypothesized that the incorporation of a precipitation inhibitor (PI) may be beneficial.

While the incorporation of PIs in a supersaturated drug delivery system has been widely explored for solid solutions, to the best of our knowledge, no study has been reported to date on the incorporation of PIs in sLBFs testing *in vivo*. A limited number of studies have explored the use of PIs to reduce the risk of drug precipitation upon the dispersion and digestion of classical LBFs.^23−26^ The principle underpinning theory of including PIs in (s)LBFs/solid solutions is commonly described by the “spring and parachute” effect.^27^ These drug delivery systems generate high initial supersaturated concentrations upon dispersion/dissolution, the so-called “spring”. To prolong precipitation from the generated supersaturated system for as long as possible, PIs act as a “parachute” by hindering nucleation and arresting precipitation.^27,28^ This fundamental advantage of prolonged supersaturated drug concentrations in the intraluminal environment is assumed to be beneficial for absorption *in vivo*.^27,29,30^ For example, Gao and co-workers demonstrated a 10-fold higher oral bioavailability by incorporating hydroxypropyl methylcellulose (HPMC) into a undersaturated LBF compared to a HPMC-free LBF in rats.^25^ Such formulations were described as “supersaturable” by Gao and colleagues, primarily referring to the ability of many LBFs to generate supersaturated drug concentrations on dispersion/digestion in intestinal fluids. However, it is worth noting that supersaturable LBFs explored by Gao et al.^23−25^ contain drugs below saturation solubility and are distinct from sLBFs even though this nomenclature is not consistently used throughout the literature. The LBFs explored in the studies by Gao and co-workers^23−25^ contained 70–100% (w/w) co-solvents and surfactants [i.e. type IV of the lipid formulation classification system (LFCS)],^31,32^ which, in general, present a greater risk of drug precipitation on dispersion. Similarly, Suys et al. recently reported that the utility of PIs to prolong supersaturation was more evident for a type IV (50% co-solvent) and type IIIB (25% co-solvent and 25% surfactants) formulation, whereas for a type IIIA formulation (no co-solvent, 35% surfactant), the PIs studied had no impact on prolonging supersaturation during *in vitro* digestion.^26^ Collectively, these studies demonstrate the merits of incorporating PIs in a LBF, where there is a perceived high risk of precipitation on dispersion/digestion due to, for example, a rapid co-solvent depletion. The aim of the present study was to address the need to study the utility of PIs in sLBFs using the model compound venetoclax.

The choice of suitable PIs, however, can be complicated^23^ as the inhibitory effect is reported to be drug specific and there are gaps in our understanding on which PIs are suited for a particular drug type.^26,27^ While various *in vitro* screening tests have been reported, to date the PI selection is mostly empirical and there is a lack of comprehensive studies compared across all the various PI types.^27^ Attempts have been made to increase the mechanistic understanding to aid with the selection of PIs to streamline formulation development;^27^ however, it is often not known how effective an *in vitro* tool is in estimating the impact on *in vivo* absorption, especially for rather complex bioenabling formulations. In addition, given the complexity of polymer–drug interactions, there is also a need for more computational tools to guide excipient selection.

Venetoclax is a highly lipophilic drug (log *P* of 5.5)^33^ with a high molecular mass of 868.44 g/mol^33^ and a melting point of 139 °C (Table S1), representing a recently licensed drug with properties in the beyond rule-of-five space. The drug is classified as a BCS class IV based on low aqueous solubility and permeability. The commercial formulation Venclyxto displays a pronounced food-dependent oral bioavailability with a 3.4-fold increase in oral bioavailability after a low-fat meal and a 5-fold increase after a high-fat meal compared to the fasted state.^33^

This study explored the merits of PIs on the oral bioavailability of an oil-based sLBF of venetoclax. A range of promising PIs have been identified based on the calculated excess enthalpy of mixing (COSMOquick software), which served to estimate the molecular excipient interaction in the more complex aqueous dispersions. The ability of the selected PIs to maintain the supersaturation of venetoclax in biorelevant media was tested *in vitro*, and the impact of incorporating PIs into the sLBF in comparison to the separate addition (pre-dissolved) of the PIs in the simulated intestinal fluids was evaluated. A subsequent *in vivo* study examined the impact of the incorporated PIs in sLBFs on the oral bioavailability of venetoclax in landrace pigs.

## Materials and Methods

### Chemicals and Materials

Venetoclax was purchased from Kemprotec Ltd. (UK) (batch # 1810004). Olive oil, highly refined and low acidity, taurodeoxycholic acid (NaTDC), and pancreatic lipase (8× USP) were ordered from Sigma-Aldrich (Ireland). Lipoid E PC S was obtained from Lipoid GmbH (Germany) and Eudragit EPO was obtained from Evonik (Germany). Hydroxypropyl methylcellulose acetate succinate (HPMCAS) [AQOAT (HPMCAS-MF)] was purchased from ShinEtsu (Japan) and Pluronic F108, HPMC, and polyvinylpyrrolidone (PVP) were purchased from MilliporeSigma (St. Louis, MO, USA). Kollidon VA 64 (PVP/VA) was kindly donated by BASF (Germany). Capmul MCM and Captex 1000 were kindly donated by Abitec Corporation (US). A sample of Peceol was kindly donated by Gattefossé (France) and SIF powder Version 1 was kindly donated by biorelevant.com (UK). Water was purified by a Milli-Q water system. All other chemicals and solvents were of analytical or high-performance liquid chromatography (HPLC) grade and were purchased from Sigma-Aldrich (Ireland) and used as received.

### Apparent Solubility

Apparent solubility was determined in olive oil, Captex 1000, Peceol, and Capmul MCM. In brief, an excess of venetoclax was added to 2 mL of the excipients and stirred at 200 rpm (25% power) (Mixdrive 15, 2MAG, Germany) at 37 °C. Solid excipients were melted at 50 °C and cooled to 37 °C prior to venetoclax addition. Samples were taken after 24, 48, and 72 h and centrifuged at 21,380*g* and 37 °C for 15 min (Mikro 200 R, Andreas Hettich GmbH & Co. KG, Germany). The supernatant was transferred to a new sample tube and centrifuged again under identical conditions. To solubilize the oily excipient, the supernatant was diluted in acetonitrile/ethyl acetate (1:3 v/v). Followed by further 1:10 (v/v) dilution with acetonitrile/ethyl acetate (3:1 v/v). The obtained samples were diluted appropriately with the mobile phase before analysis by reverse-phase HPLC as described below. All samples were run in triplicates.

### Biorelevant Solubility

FaSSIF and fed state simulated intestinal fluid (FeSSIF) was prepared according to the instructions by biorelevant.com. FeSSIF was used directly, whereas FaSSIF was left at room temperature for 2 h prior to usage. Excess venetoclax was added to 2 mL of biorelevant media and placed in a water bath shaker at 200 shakes/min (GLS400, Grant Instruments, UK) and 37 °C. Samples were taken after 3, 6, and 24 h and centrifuged at 21,380*g* and 37 °C for 15 min (Mikro 200 R, Andreas Hettich GmbH & Co. KG, Germany). The supernatant was transferred to a new sample tube and centrifuged again under identical conditions. Subsequently, the supernatant was diluted with the mobile phase before analysis by HPLC.

The samples were analyzed using an Agilent 1200 series HPLC system (Agilent Technology Inc., US) that comprised a binary pump, degasser, autosampler, and variable wavelength detector. Data were analyzed using the software EZChrom Elite version 3.2. Venetoclax was separated from the sample matrix with a Zorbax Eclipse Plus-C18 column (5 μm, 4.6 mm × 150 mm) including a Zorbax Eclipse Plus-C18 guard column (5 μm, 4.6 mm × 12.5 mm) at 40 °C. The mobile phase consisted of (a) acetonitrile with 0.5% trifluoroacetic acid (TFA) and (b) water with 0.5% TFA at a ratio of 53:47 (a/b v/v) and was used at a flow rate of 1 mL/min. The injection volume was 20 μL and the detection wavelength was set to 316 nm. The limit of detection was 20 ng/mL and the limit of quantification (LOQ) was 65 ng/mL determined using the standard error of the *y*-intercept according to the International Council for Harmonization (ICH) Q2 guidelines.^34^

### Formulations for *In Vivo* and *In Vitro* Studies

In the *in vivo* study and *in vitro* PI screens, the supersaturated lipid solution (sLBF) was prepared as previously reported with a lower temperature to reduce the thermal impact on the drug and excipient.^13^ In brief, 300 mg of venetoclax were added to 6 mL of Peceol (50 mg/mL) and dispersed at 600 rpm (Stuart CD162 heat-stir, Cole-Parmer, UK) and sealed with parafilm. A continuous nitrogen stream into the vial removed oxygen throughout the preparation. After suspending the drug particles, the obtained suspension was slowly heated to 55 °C (Stuart CD 162 heat-stir, Cole-Parmer, UK). The mixture was kept at 55 °C for 10 min and cooled to 25 °C while continuously stirring at 600 rpm. Subsequently, the mixture was heated a second time under the same conditions as stated above and cooled to room temperature to obtain the final sLBF. The absence of crystals was confirmed using cross-polarized light microscopy. For the *in vivo* study, sLBF was administered in hard gelatin capsule size 00EL (Licaps, Capsugel, Lonza Group Ltd.) with 1 mL/capsule.

For the preparations of the sLBF with PI, HPMC, HPMCAS, Pluronic F108, Eudragit EPO, PVP, and PVP-*co*-vinyl acetate (PVP/VA) were added to the sLBF at a drug/PI ratio of 1:1 (w/w). At the given PI concentration (50 mg/mL), Pluronic F108, Eudragit EPO, PVP, and PVP/VA were soluble in Peceol at 37 °C, while HPMC and HPMCAS resulted in a suspension. In the case of the soluble PIs, the Peceol-PI solution was used to prepare the PI containing sLBF using the method described above. In the case of HPMC and HPMCAS, the sLBF was prepared with Peceol as described above, and HPMC and HPMCAS were added as the powder and dispersed ad hoc into the sLBF before *in vitro* and *in vivo* experiments. The amount of PI in the lipid vehicle (50 mg/mL) was based on a previous work by Gao et al.^23,24^

### Viscosity of Venetoclax Containing sLBFs

The viscosity of the venetoclax containing sLBF and sLBF with PIs was measured using a rotational viscometer (Discovery HR-1, TA Instruments, USA) with a 60.0 mm plate and a 2.0° cone (Peltier plate titanium, solvent trap, TA Instruments, USA). The data were analyzed using a Trios V5.1.0.46403 (TA Instruments, USA). Viscosity measurements were performed at 37 °C by pre-heating the formulations to 37 °C in a heating cabinet and by allowing for a temperature equilibration time of 180 s prior to commencing the viscosity measurements. The utilized method constituted three steps. First, a shear versus viscosity curve was obtained by increasing the shear rate stepwise (10 measurement points) from 0.0 to 600 1/s over 300.0 s (30 s/measurement point). Second, the shear rate was set to 0.0 1/s for 60 s. Finally, the shear rate was set to 300 1/s for 60 s with a sampling interval of 6.0 s/measurement point.

### Cross-Polarized Light Microscopy

The absence of the crystalline material in the supersaturated solutions was confirmed by means of cross-polarized light microscopy using an Olympus BX51 with an Olympus SC100 camera operated by Olympus Stream essentials 2.3.3 (Olympus, UK). The light was polarized using the polarizer U-POT (Olympus, UK) and analyzed with the analyzer U-ANT (Olympus, UK). The absence of crystals was assumed, if no birefringence was observed.

### *In Vitro* Evaluation: Drug Solubilization during Formulation Dispersion and Digestion

*In vitro* lipolysis was performed using a pH-stat apparatus (Metrohm AG, Herisau, Switzerland) comprising a Titrando 907 stirrer, 804 Ti-stand, a pH electrode (Metrohm AG, Herisau, Switzerland), and two 800 Dosino dosing units coupled to a 20 mL autoburette. The system was operated by the Tiamo 2.2 software. The *in vitro* protocol was used as previously reported.^35^ In brief, the buffer contained 2 mM TRIS maleate, 150 mM NaCl, and 1.4 mM CaCl_2_·2H_2_O, adjusted to pH 6.5. For the digestion experiments, the buffer was supplemented with 3 mM NaTDC and 0.75 mM PC (digestion buffer) and stirred for 12 h before further usage. The pancreatin extract was prepared freshly by adding 5 mL of 5 °C digestion buffer to 1 g of porcine pancreatic enzymes (8× USP), which was vortexed thoroughly. The mixture was centrifuged for 15 min at 5 °C, 2800*g* (Rotina 380 R, Andreas Hettich GmbH & Co. KG, Germany), and 4 mL of the supernatant was recovered and stored at 2–8 °C before further usage. The pancreatic extract had a pancreatic lipase activity of ∼10,000 TBU/mL (to provide approximately 1000 TBU per mL of digest), where 1 TBU represents the amount of the enzyme that liberates 1 μmol of FA from tributyrin per min.^36^

For the *in vitro* lipolysis experiment, 1.075 g of the lipid formulation was dispersed into 39 mL of digestion buffer for 10 min. Three 1 mL samples were taken at 2.5, 5, and 10 min from the middle of the vessel. pH of the media was adjusted and maintained at 6.5 using 0.2 M NaOH. To the remaining 36 mL (1.0 g lipid formulation) of the dispersion, 4 mL of the pancreatin extract was added to initialize digestion. After 60 min, the released nonionized free fatty acids were determined by a pH increase of the buffer to pH 9. The stirring speed throughout dispersion and digestion was set at 450 rpm.

Samples of 1.0 mL were taken at 5, 10, 15, 30, 45, and 60 min during the digestion experiment from the middle of the vessel. In each sample and after 60 min, the enzymes were inhibited by the addition of 1 M 4-bromophenylboronic acid in methanol (5 μL per mL sample). Additionally, to each 1 mL sample during digestion a 100 μL sample was taken and added to 900 μL of acetonitrile and mixed. This sample was used to quantify the total drug recovery, which allowed the adjustment of inhomogeneous samples. All samples were centrifuged at 37 °C and 21,000*g* for 30 min using a benchtop centrifuge of the type Hettich Micro 200 R (Andreas Hettich GmbH & Co. KG, Germany).

### *In Silico* PI Screening: COSMO-RS Calculations

The excess enthalpy of mixing between venetoclax and the polymeric PI was calculated using COSMOquick software (COSMOlogic, Germany, Version 1.6). This software is based on the conductor-like screening model for real solvents^37,38^ that combined quantum chemical surface charge calculations with statistical thermodynamics. The COSMOquick approach in particular allows for a fast calculation of surface charge densities based on molecular fragments of previously calculated compounds.^39^ Venetoclax and the polymers were entered in smiles notation. As the quantum chemical calculations cannot capture the full complexity of the polymers, such macromolecules have to be approximated and this study used trimers for this purpose as previously described by Price et al. for supersaturating formulations.^40^ The drug/PI ratio was set at a stoichiometric ratio of 1:1 to represent the formulations used *in vitro* and *in vivo* and the temperature was set to 37 °C. In line with previous applications of COSMOquick for co-former screening in co-crystal selection,^41^ drug solubility estimations in glycerides,^42^ and polymer screening for supersaturated formulations,^40^ a more negative value of the calculated excess enthalpy ranks the strength of molecular drug–excipient interaction. This is just an approximation, as the presence of an aqueous phase is not considered in the calculations nor the complexity of any biorelevant medium. Therefore, the results should be understood as a first *in silico* estimation of relative excipient comparison. This has previously been referred to as a higher or lower “COSMO-Rank”.^40^

### *In Vitro* PI Testing

The *in vitro* PI testing for venetoclax was done by means of a solvent shift. The effect of a fully hydrated PI (dissolved in FaSSIF) was compared against the PI in the formulation. Thus, the employed PIs HPMC, HPMCAS, Eudragit EPO, PVP, PVP/VA, and Pluronic F108 were dissolved in either FaSSIF or either suspended or dissolved in the sLBF. For the hydrated PI test, 5 mg of PI was dissolved in 5 mL of FaSSIF [prepared according to biorelevant.com) and 5 mg of venetoclax dissolved in either DMSO (100 mg/mL) or Peceol (sLBF 50 mg/mL] was added. For the evaluation of the PI in the lipid formulation, 100 μL of the sLBF (50 mg/mL venetoclax and 50 mg/mL PI) was added to 5 mL of FaSSIF. The vials were sealed and placed in a water bath shaker at 200 shakes/min and 37 °C (GLS400, Grant Instruments, UK). After 2, 5, 10, 15, 30, 60, 120, and 180 min, 250 μL samples were taken. The samples were filtered with a 0.2 μm syringe filter (Whatman Spartan 13/0.2) and samples that contained lipids were additionally centrifuged for 30 min at 21,380*g* and 37 °C (Mikro 200 R, Hettich GmbH, Germany). The aqueous phase was collected, and one part was diluted 1:10 (v/v) with acetonitrile (including 0.5% (v/v) TFA). A pure (nondiluted) and a diluted sample were analyzed by HPLC as described above.

Additionally, the solubility of venetoclax was determined in FaSSIF with dissolved PI. Excess venetoclax was added to 5 mL of FaSSIF with 5 mg of PI. 250 μL of samples were taken at 3, 6, and 24 h and filtered through a 0.2 μm syringe filter (Whatman Spartan 13/0.2), diluted with acetonitrile (containing 0.5% (v/v) TFA), and analyzed by HPLC as described above. Apparent supersaturation ratios (SR_app_) were calculated for each formulation based on the obtained FaSSIF solubility and the measured venetoclax concentration in the 5 min sample of the *in vitro* PI testing according the following formula
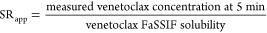


### Evaluation of the sLBF Dispersion: Zeta Potential and Droplet Size Analysis

sLBFs containing venetoclax were prepared and dispersed in FaSSIF (at 37 °C) as described above (1:50 (v/v) dilution). Samples (250 μL) were taken after 5 and 60 min and diluted 1:250 (v/v) with FaSSIF V1. The diluted samples were analyzed for its micellar/droplet size and zeta potential using a Malvern Nano-Zetasizer (Malvern Panalytical Ltd., UK). For the dynamic light scattering analysis of the micellar/droplet size, the samples (1 mL) were measured using disposable ZEN0040 cuvettes and the micelles/droplet size was recorded according to the intensity-based size distribution. Measurements were done in triplicates at 37 °C, using 3 measurements with 12 runs of 10 s each. The measurement angle was 173°. The dispersant medium was customized as FaSSIF buffer containing the buffer salts and sodium chloride (according to the concentrations in FaSSIF V1)^43^ with a refractive index of 1.332 and a viscosity of 0.7252 cP. The material absorption was set to 0.01 and the material refractive index to 1.44. The zeta potential was measured using a folded capillary cell (DTS1070). Measurements were performed in triplicate, at 37 °C, using 3 measurements with 12 runs of 10 s each.

### *In Vivo* Study

All the experiments were approved and conducted with licenses issued by the Health Products Regulatory Authority, Ireland (project license AE19130/P058) as directed by the EU Statutory instruments of the EU directive 2010/63/EU (Protection of Animals used for Scientific Purposes). Local ethical approval was granted by University College Cork Animal Experimentation Ethics Committee (AEEC). In order to test all PIs, two bioavailability studies had to be conducted. The first study was with 5 pigs and a 6-way cross-over and the second study with 3 pigs and a 4-way cross-over. Both studies were randomized and conducted in male landrace pigs (15–17 kg) and each pig received a single dose of 100 mg of venetoclax. Pigs were fed approximately 175 g of the standard weanling pig pellet feed twice daily. In the fasted study legs, the final feed of 175 g was given 24 h prior to dosing. As part of the study design, any remaining food was removed 16 h before dosing; however, no food remained at this point in any of the groups. On day 1, an indwelling intravenous catheter was inserted from the ear vein into the jugular vein under general anesthesia, which was used for repeated blood sampling throughout the study. In the first study, on day 3, following an overnight fast of 16 h, the pigs were administered either sLBF or sLBF with PI, respectively. The tested PIs included HPMC, HPMCAS, PVP, PVP/VA, and Pluronic F108. In the second study, on day 3, following an overnight fast of 16 h, pigs were administered either a reference capsule with venetoclax powder, a venetoclax Peceol suspension, a sLBF, or a sLBF including Eudragit EPO, respectively. The results of the venetoclax powder, Peceol suspension, and sLBF have previously been reported^13^ and are not further described in the current study. In both studies, all formulations were administered with the aid of a dosing gun, followed by 50 mL of water via a syringe. In order to control the water intake with the dosage forms, the water availability was restricted for 3 h postdosing. At all other times, water was available *ad libitum*. To facilitate handling during the oral administration, an intramuscular dose of ketamine (5 mg/kg) and xylazine (1 mg/kg) was administered in both studies. Blood samples were collected after 0.5, 1, 1.5, 2, 3, 4, 5, 6, 7, 8, 9, 10, 12, and 24 h in heparinized tubes. Upon collection, blood samples were immediately centrifuged at 3000*g*, 4 °C for 5.5 min (Eppendorf 5702 R, Rotor A-4-38, Eppendorf Ltd., UK). The supernatant plasma was harvested and stored at −20 °C until further analysis. A 6-day washout period was maintained between the study legs.

### Bioanalysis

The plasma concentrations of venetoclax were determined by reversed-phase HPLC. The Agilent 1260 series HPLC system (Agilent Technology Inc., US) comprised a binary pump, degasser, temperature controlled autosampler, column oven, and diode array detector. The system was operated, and the data analyzed with EZChrom Elite version 3.3.2. A Zorbax Eclipse Plus-C18 column (5 μm, 4.6 mm × 150 mm) with a Zorbax Eclipse Plus-C18 guard column (5 μm, 4.6 mm × 12.5 mm) were used for the separation of venetoclax. The mobile phase consisted of water and acetonitrile with 0.5% (v/v) TFA at a ratio of 47:53 (v/v) and was used at a flow rate of 1.0 mL/min. The sample and column temperature were set at 5 and 40 °C, respectively, and the detection wavelength was set to 250, 290, and 316 nm. Venetoclax was extracted from the plasma samples by liquid–liquid extraction as reported previously.^13^ In brief, venetoclax was extracted from 500 μL of the plasma using acetonitrile and ethyl acetate. Vemurafenib was used as the internal standard. The extraction solvents were dried under a nitrogen stream at 60 °C, and the residues were reconstituted in 100 μL of the mobile phase (excluding TFA), followed by centrifugation at 25 °C, 11,500*g* for 5 min (Mikro 200 R, Andreas Hettich GmbH & Co. KG, Germany). The injection volume used for HPLC analysis of the supernatant was 50 μL.

### Data Analysis

Prior to statistical analysis, the Bartlett’s test was used to check for equal variances. A one-way analysis of variance (one-way ANOVA) was performed for the lipolysis and *in vitro* PI test data as well as the area under the curve (AUC) of the *in vitro* PI test using Tukey’s post-hoc test to compare the different formulation performances. The pharmacokinetic parameters were calculated using Microsoft Excel by means of the trapezoidal rule. The plasma concentration profiles were analyzed by noncompartmental analysis. The statistical analysis for the *in vivo* parameters of the formulations within the same cross-over study was performed using a one-way ANOVA after using the Bartlett’s test to check for equal variances. The pairwise comparison of the groups was done using Tukey’s multiple comparison test. All statistical analyses were carried out using Prism version 5 by GraphPad.

## Results

### Apparent Solubility of Venetoclax in Lipid Excipients

**Figure 1:**
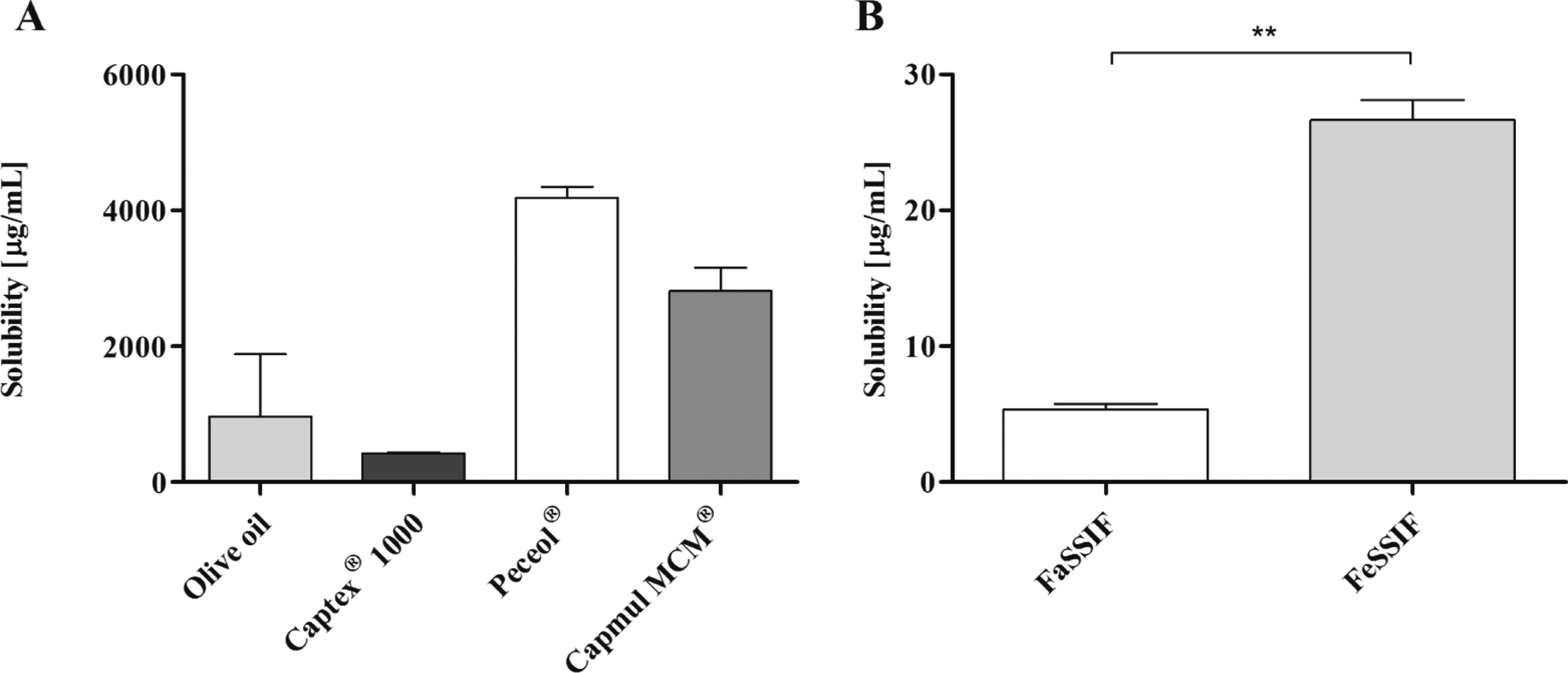
(A) Venetoclax solubility in lipid excipients at 37 °C (*n* = 3, mean ± SD), (B) venetoclax solubility in FaSSIF and FeSSIF at 37 °C (*n* = 3, mean ± SD).

Solubility screening in the pure lipid excipient indicated that venetoclax showed a higher apparent solubility in the long chain than the medium chain-based excipients (Figure 1A). Within the long chain and medium chain excipients, a higher apparent solubility was observed for the monoglycerides compared to the triglycerides. Subsequently, the biorelevant solubility of venetoclax was determined (Figure 1B). Solubility in FaSSIF was 6.8 ± 1.8 μg/mL translating to only 1.7% of a 100 mg dose venetoclax in 250 mL of the gastrointestinal fluid. In the fed state, the solubility increased 3.9-fold to 6.7% of the 100 mg of venetoclax dose, which in general is in agreement with the observation in increased bioavailability for venetoclax reported from clinical studies.^33^

### sLBF Achieves High Aqueous Drug Concentration during *In Vitro* Dispersion and Digestion

Previous studies by our group developed a venetoclax sLBF using a preparation method at 70 °C.^13^ Process optimization in this study focused on improving the thermally induced supersaturation process by lowering the overall energy input while maintaining the desired level of supersaturation. sLBFs were prepared at a target dose load of 50 mg/mL by heating a LBF suspension (50 mg/mL) to 55 °C. The (s)LBF was kept at 55 °C for 10 min to dissolve venetoclax before cooling the solution to room temperature. In this study, the obtained sLBF had a 11.9-fold (1193.7%) higher drug load compared to the apparent saturation solubility. The venetoclax concentrations in the aqueous phase during dispersion and digestion are shown in Table 1 and Figure S1.

**Table 1:** Venetoclax Concentration in the *In Vitro* Lipolysis Experiment after 10 min of Dispersion and after 5 and 60 min of Digestion^a^

	venetoclax concentration (μg/mL) in aqueous phase (AP) during LBF dispersion and digestion		
formulation	AP_dispersion_ (10 min)	AP_digestion_ (5 min)	AP_digestion_ (60 min)
sLBF^b^	18.7 ± 0.0	37.5 ± 3.2	73.8 ± 6.4
LBF suspension^c^^,^^d^	1.0 ± 0.1	1.7 ± 0.3	2.9 ± 0.5
aqueous suspension^d^	3.0 ± 0.1	9.1 ± 0.2	9.4 ± 0.3

a Venetoclax was formulated as sLBF (50 mg/mL), Peceol suspension (50 mg/mL), and aqueous suspension (50 mg/mL). All the experiments were run with *n* = 3 and results are shown as mean ± SD.

b sLBF drug loading was 1194% of determined apparent solubility.

c LBF drug loading was 100% solubilized + 158% suspended of determined apparent solubility.

d Data as previously reported.^13^

As the drug needs to be dissolved prior to absorption by the body, the solubilized drug in the aqueous phase of the *in vitro* lipolysis test is often used as a guide to formulation performance *in vivo*. The sLBF displayed relatively high concentrations in the aqueous phase throughout *in vitro* lipolysis, with an average venetoclax concentration of 18.6 ± 1.3 μg/mL during dispersion and 73.8 ± 6.4 μg/mL after 60 min of digestion. Interestingly, the final concentration of the digestion (60 min) was equivalent to 6.4 ± 0.5% of the venetoclax dose used in the experiment and was 2.8-fold higher when compared to the apparent solubility of venetoclax in FeSSIF. Additionally, the measured concentration was higher than the reported amorphous solubility in FeSSIF of 26.4–54.6 μg/mL.^44^ Most of venetoclax was recovered in the lipid phase with an average of 98.5 ± 2.8% of the venetoclax dose during dispersion and 88.6 ± 0.2% after 60 min of digestion (Figure S1). The lipid phase can act as a reservoir for the drug, which gradually diffuses into the aqueous phase during the transit through the gastrointestinal tract. Additionally, the lipid phase is digested during the transit releasing venetoclax. In the case of the sLBF, the lowest amount of venetoclax was recovered in the solid phase. sLBF showed no precipitation during dispersion and after 60 min of digestion only 6.2 ± 0.6% of the venetoclax dose was precipitated. These results are in line with previous studies showing a superior performance (a high-aqueous-phase concentration and a low-solid-phase concentration) of the sLBF *in vitro* relative to a lipid-based suspension and an aqueous suspension.^13^ The optimized processing method, therefore, yielded a comparable formulation performance.

### COSMO-RS as a Preliminary *In Silico* Screening Tool for PI Selection

**Figure 2:**
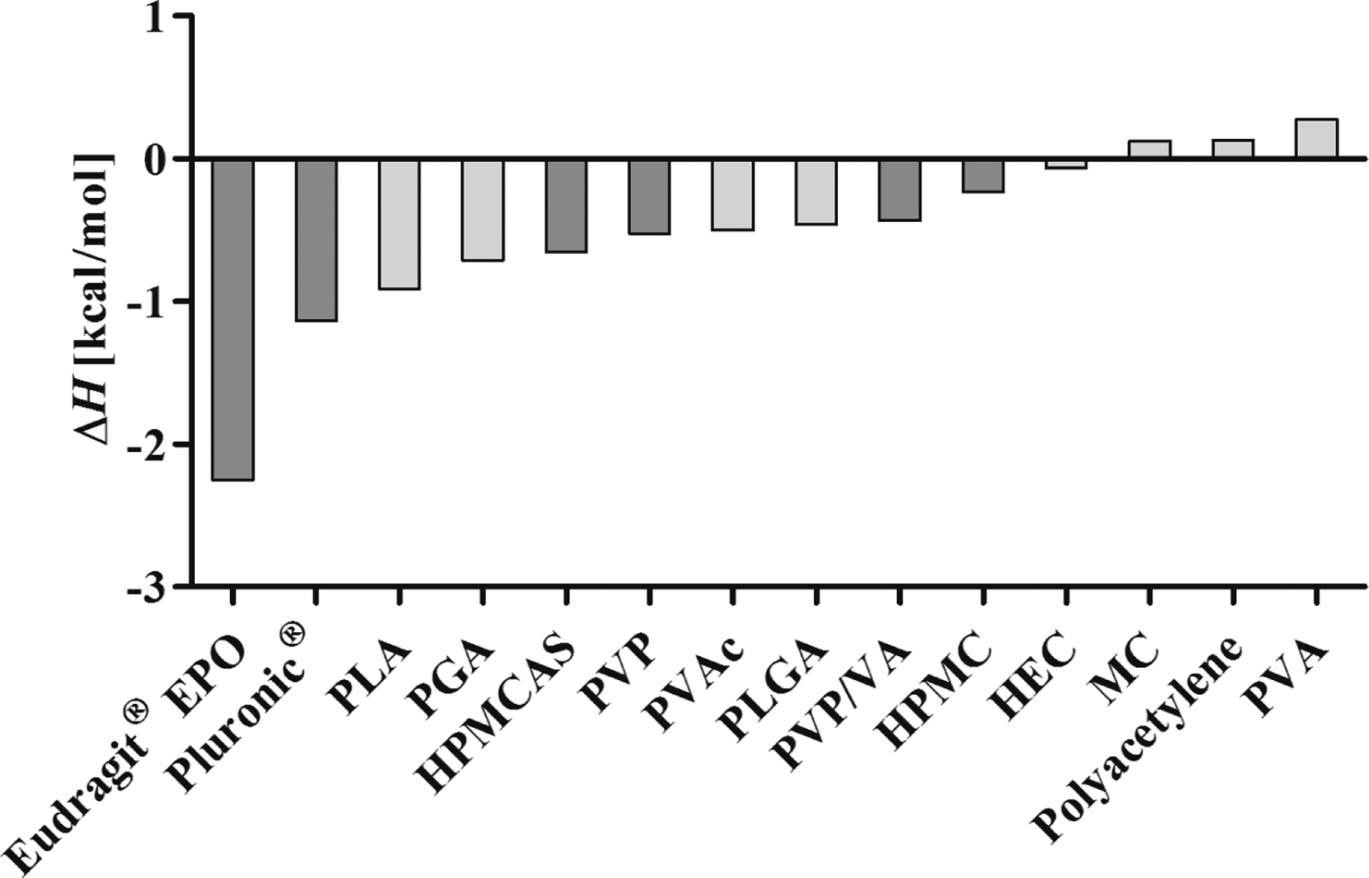
COSMOquick screen. Calculated excess enthalpy of interaction between venetoclax and the PIs. The dark gray PIs were selected for further *in vitro* and *in vivo* evaluation. The calculation was based on a 1:1 ratio of drug to PI. PLA: polylactic acid, PGA: polyglycolic acid, HPMCAS: hydroxypropyl methylcellulose acetate succinate, PVP: polyvinylpyrrolidone, PVAc: polyvinyl acetate, PLGA: polylactic-*co*-glycolic acid, PVP/VA: polyvinylpyrrolidone-*co*-vinyl acetate, HPMC: hydroxypropyl methylcellulose, HEC: hydroxypropyl cellulose, MC: methylcellulose, and PVA: polyvinyl alcohol.

To identify the most promising PIs, reduce the extent of *in vitro* testing, and to evaluate the utility of this computational tool in the formulation design of sLBFs, the interactions between venetoclax and various PIs were evaluated *in silico*. Based on the COSMO-RS theory^37,45^ that combines quantum chemistry and statistical thermodynamics, COSMOquick software was utilized to predict binary PI–drug interactions similarly to previous reports, where this screening technique of PI selection was utilized for silica formulations.^40^ COSMOquick software predicts the interactions of two molecules based on equilibrium thermodynamics and the predicted chemical potential in liquids. The stronger the interaction between venetoclax and the PI, the more negative the calculated excess enthalpy of interaction. A simplified view assigns to a PI that has a more negative excess enthalpy, a higher “COMSO-Rank” with respect to its suitability as PI. While this tool may be beneficial to reduce *in vitro* testing by identifying the most promising PI–drug interactions as shown for other formulation approaches,^40^ it must be acknowledged that the *in silico* calculations did not consider the presence of water nor other formulation excipients, potentially limiting the applicability of this approach for the more complex bioenabling formulation approach of LBFs. In the present study, the excess enthalpy of mixing for a variety of PIs was calculated, and PIs with varying negative enthalpy of mixing were selected. The chosen PIs had the following “COSMO-Rank”: Eudragit EPO > Pluronic > HPMCAS ≥ PVP ≥ PVP/VA > HPMC as shown in Figure 2.

### *In Vitro* Testing of PIs for Prolonging Supersaturation

**Figure 3:**
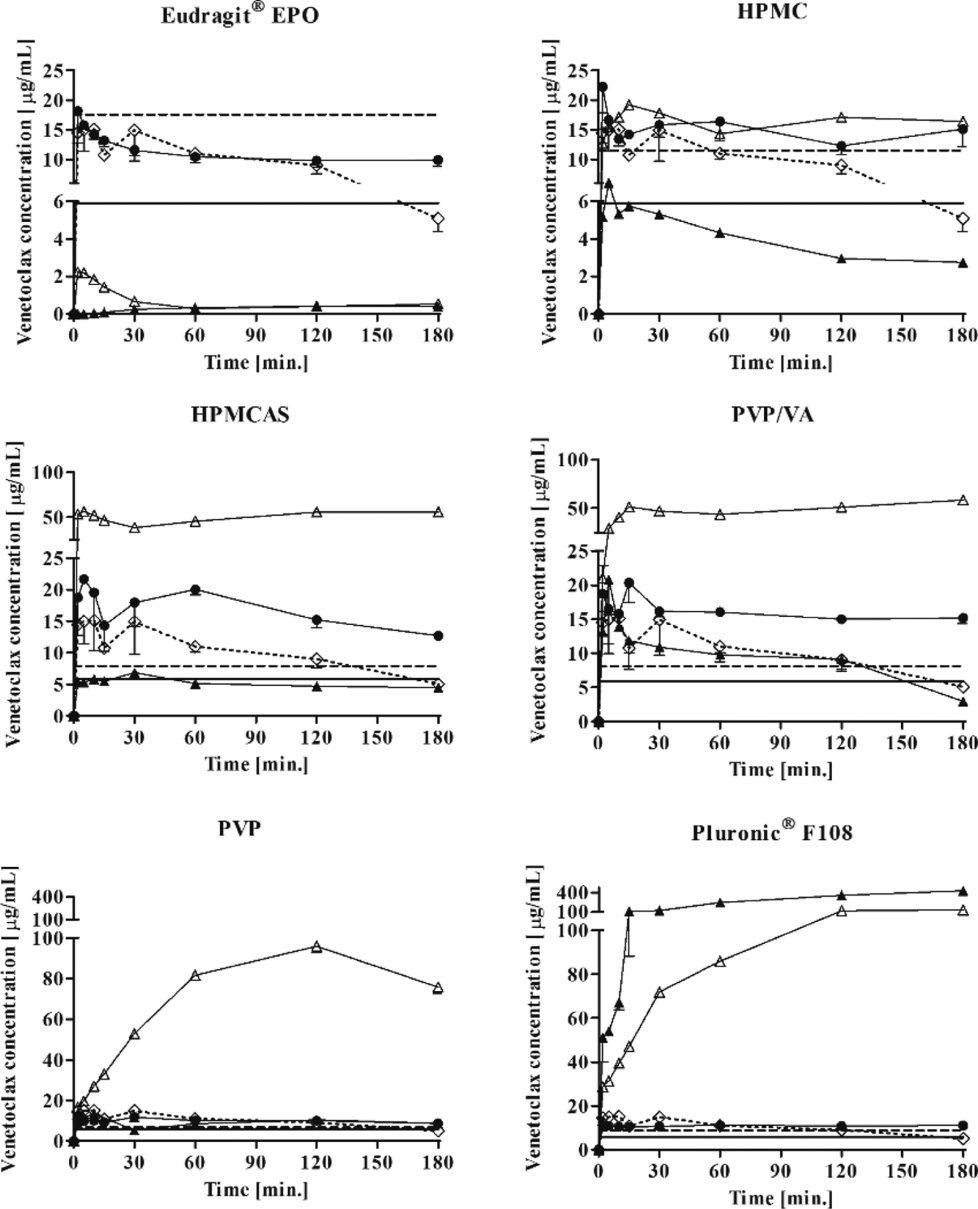
*In vitro* evaluation of the venetoclax concentration profile during the dispersion of sLBFs in FaSSIF (mean ± SD with *n* = 3). The dashed line represents the apparent venetoclax solubility in FaSSIF with pre-dissolved PI (FaSSIF-PI) and the solid line represents FaSSIF solubility. The dotted line (◊) represents the sLBF alone dispersed in FaSSIF (sLBF-noPI). Venetoclax dissolved in DMSO dispersed in FaSSIF-PI (DMSO control) (●), sLBF dispersed in FaSSIF-PI (sLBF-aqPI) (▲), and sLBF with PI (sLBF-PI) dispersed in FaSSIF (Δ).

The selected PIs from the *in silico* screening were further evaluated experimentally *in vitro* to assess the PI performance (aqueous phase concentration) under simulated conditions and with LBFs. The *in vitro* PI test was performed by means of the solvent shift by dispersing the formulation in (i) FaSSIF or (ii) FaSSIF with pre-dissolved PI (FaSSIF-PI). In FaSSIF-PI was dispersed (a) venetoclax dissolved in DMSO (100 mg/mL) as positive control (DMSO control) and (b) sLBF (sLBF-aqPI). In FaSSIF was dispersed (c) sLBF without PI (sLBF-noPI) and (d) sLBF with incorporated PI (sLBF-PI). Polymers that were soluble at the tested concentration of 50 mg/mL were dissolved in sLBF (PVP, PVP/VA, Pluronic F108, Eudragit EPO) and those that were not soluble in the sLBF were therefore ad hoc suspended in the sLBF (HPMC, HPMCAS). The concentration of PI in the sLBF was based on previous reports by Gao et al.^23,24^ Additionally, the solubility of venetoclax in FaSSIF-PI was measured. The apparent supersaturation of the formulations was calculated using the venetoclax concentration after 5 min of dispersion, relative to the FaSSIF solubility. The apparent supersaturation ratio indicates the apparent fold increase in the venetoclax concentration above the FaSSIF solubility at the given time point. The results of the PI testing are shown in Figure 3 and Table 2.

**Table 2:** AUC of the *In Vitro* PI Testing for sLBF Dispersed in FaSSIF-PI (sLBF-aqPI), sLBF with PI Incorporated in the Formulation (sLBF-PI) and Dispersed in FaSSIF (FaSSIF-PI), Venetoclax Dissolved in DMSO and Dispersed in FaSSIF-PI (DMSO control), and sLBF Alone Dispersed in FaSSIF (sLBF-noPI); % Solubilized Venetoclax and Apparent Supersaturation Ratio for sLBF-aqPI and sLBF-PI as Well as Apparent FaSSIF-PI Solubility (Mean ± SD, *n* = 3)

	AUC [mg·min/mL]				solubilized venetoclax [%]^a^		apparent supersaturation ratio^b^	
PI	DMSO control	sLBF-aqPI	sLBF-PI	apparent FaSSIF-PI solubility [μg/mL]	sLBF-aqPI	sLBF-PI	sLBF-aqPI	sLBF-PI
Eudragit EPO	1.9 ± 0.3	0.1 ± 0.004	0.1± 0.02	17.6 ± 0.8	0.03 ± 0.002	0.1 ± 0.01	<LOQ	0.3 ± 0.03
HPMC	2.6 ± 0.5	0.7 ± 0.1	2.9± 0.3	11.5 ± 2.4	0.4 ± 0.04	1.6 ± 0.2	0.9 ± 0.2	2.3 ± 0.2
HPMCAS	3.0 ± 0.3	0.9± 0.03	9.1 ± 0.4	7.9 ± 1.6	0.5 ± 0.02	5.1 ± 0.2	0.8 ± 0.1	8.3 ± 0.3
PVP	1.8 ± 0.3	1.5 ± 0.2	13.5 ± 0.1	6.7 ± 0.2	0.9 ± 0.1	7.5 ± 0.04	1.7 ± 0.2	2.9 ± 0.4
PVP/VA	2.8 ± 0.2	1.6 ± 0.2	8.8 ± 0.3	8.1 ± 1.4	0.9 ± 0.1	4.9 ± 0.2	3.0 ± 1.6	4.3 ± 0.3
Pluronic F108	2.0 ± 0.4	50.4 ± 2.4	17.5 ± 0.6	8.9 ± 0.2	28.1 ± 1.3	9.8 ± 0.3	7.9 ± 0.2	4.6 ± 0.2
sLBF-noPI	1.8 ± 0.3				1.0 ± 0.2		2.2 ± 0.5	

a % solubilized was calculated by dividing the AUC of the concentration versus time profiles by the maximum AUC, that is, representing 100% solubilized, over the same period of time.

b Apparent supersaturation ratio (SR_app_) of venetoclax after 5 min dispersion (SR_app_ = determined venetoclax concentration/venetoclax FaSSIF solubility).

In all cases, the venetoclax solubility in FaSSIF-PI was higher compared to the solubility in FaSSIF with an increase in solubility from 1.3-fold to 3.4-fold. In order of decreasing solubility in FaSSIF-PI, the ranking was Eudragit EPO, HPMC, Pluronic F108, PVP/VA, HPMCAS, and PVP. In general, upon the dispersion of the DMSO solution in FaSSIF-PI HPMC, Pluronic F108, PVP/VA, HPMCAS, and PVP, venetoclax concentrations reached FaSSIF-PI solubility, which was maintained throughout the experiment. In the case of Eudragit EPO, a decrease below the FaSSIF-PI solubility was observed.

In the case of the sLBF-noPI, a high initial concentration was observed (14.5 ± 1.7 μg/mL), which was above the FaSSIF solubility (5.2 ± 0.4 μg/mL). In addition, the initial venetoclax concentrations of sLBF-noPI was above FaSSIF-PI in the case of HPMC, PVP/VA, HPMCAS, PVP, and Pluronic F108, indicating that the investigated PI-free sLBF-noPI was able to generate supersaturation, that is, a spring effect upon dispersion. The observed initial spring effect venetoclax concentrations of the sLBF-noPI were also similar to all the initial aqueous phase concentrations of DMSO control, which had the PIs pre-dissolved in the media. Relative to the FaSSIF solubility, an apparent supersaturation ratio of 2.2 ± 0.5 after 5 min of dispersion was observed for the sLBF-noPI (Table 2). The degree of supersaturation from the sLBF-noPI gradually decreased to 1.3 ± 0.2 after 2 h, whereas at 3 h, the drug concentration had dropped to FaSSIF solubility. However, while the sLBF-noPI was not able to maintain supersaturated concentrations throughout the experiment, the use of PIs in the positive control maintained supersaturation for up to 3 h. As the function of the PIs was intended as a “parachute” to prolong supersaturation, the results indicated a beneficial precipitation inhibitory effect of PIs on the formulation performance.

Interestingly, the combination of PIs in sLBF (sLBF-PI) yielded a higher spring concentration in the case of HPMCAS, PVP, PVP/VA, and Pluronic F108 when compared to sLBF-noPI and DMSO control. While the sLBF-PI with HPMC showed an initial venetoclax concentration similar to sLBF-noPI, sLBF-PI with Eudragit EPO resulted in a statistically significant lower venetoclax concentration in the media when compared to sLBF-noPI and DMSO control (*p* < 0.05). In fact, the venetoclax concentration was below FaSSIF and FaSSIF-PI solubility when Eudragit EPO and lipid were present simultaneously. In the case of dispersing sLBF into FaSSIF-PI (sLBF-aqPI), an increased initial venetoclax concentration was only evident for Pluronic F108 when compared to sLBF-noPI. All other PIs resulted in a similar or decreased initial concentration. However, the increased venetoclax concentration in the presence of Pluronic F108 might also be attributed to the additional solubilization effects of Pluronic F108 as a non-ionic surfactant.

The impact of polymer-sLBF combinations on the ability to solubilize venetoclax and prolong supersaturation across six different PIs is summarized in Table 2 and Figure 3. In the case of HPMC, HPMCAS, and Eudragit EPO, the sLBF-aqPI resulted in a lower amount of solubilized venetoclax throughout the experiment compared to sLBF-noPI. Similarly, in comparison to DMSO controls, the sLBF-aqPI resulted in a lower AUC in the case of Eudragit EPO, HPMC, HPMCAS, and PVP/VA. As an example, the amount of the solubilized venetoclax dose was 0.033 ± 0.002% for Eudragit EPO in the case of sLBF-aqPI. In addition, sLBF-aqPI resulted in the case of Eudragit EPO, HPMC, HPMCAS, PVP, and PVP/VA in a similar or lower apparent supersaturation ratio compared to sLBF-noPI and the respective DMSO controls. In the case of Pluronic F108, the amount of venetoclax solubilized and the apparent supersaturation ratio were increased compared to the sLBF-noPI and DMSO control. This may be attributed to the surfactant properties of Pluronic F108, which can lead to an improved dispersibility of the sLBF and higher solubility of venetoclax. Overall, the results of the sLBF-aqPI suggested that a pre-dissolved PI in FaSSIF was less effective at prolonging venetoclax supersaturation, indicating that concomitant administration (i.e., chase dosing) a PI with a sLBF would not potentially offer benefits in terms of the sustained high concentrations for absorption *in vivo*.

In the case of incorporating HPMC, HPMCAS, PVP, PVP/VA, and Pluronic F108 within the sLBF a higher amount of venetoclax was solubilized compared to sLBF-noPI. Furthermore, the sLBF-PIs resulted in a higher amount of solubilized venetoclax in comparison to the sLBF-aqPI. In addition, higher apparent supersaturation ratios (after 5 min of dispersion) were observed for sLBF-PI relative to sLBF-noPI. In the case of HPMC, HPMCAS, PVP, and PVP/VA, the apparent supersaturation ratios were higher compared to the sLBF-aqPI, indicating that these sLBF-PI combinations achieved a balance between generating and maintaining supersaturated concentrations. However, Pluronic F108 and Eudragit EPO did not follow this trend. Pluronic F108 showed a higher supersaturation ratio and % solubilized venetoclax for the sLBF with pre-dissolved Pluronic F108 (sLBF-aqPI), compared to the incorporation into the sLBF (sLBF-PI). Such high venetoclax concentrations and supersaturation ratios in the presence of Pluronic F108 may be attributed to the surfactant nature of the polymer, which can increase the solubilization capacity of the test media, especially when pre-dissolved. In the case of Eudragit EPO, a significantly decreased amount of solubilized venetoclax and supersaturation ratios <1 in all cases of lipid excipient-PI combinations were observed.

**Figure 4:**
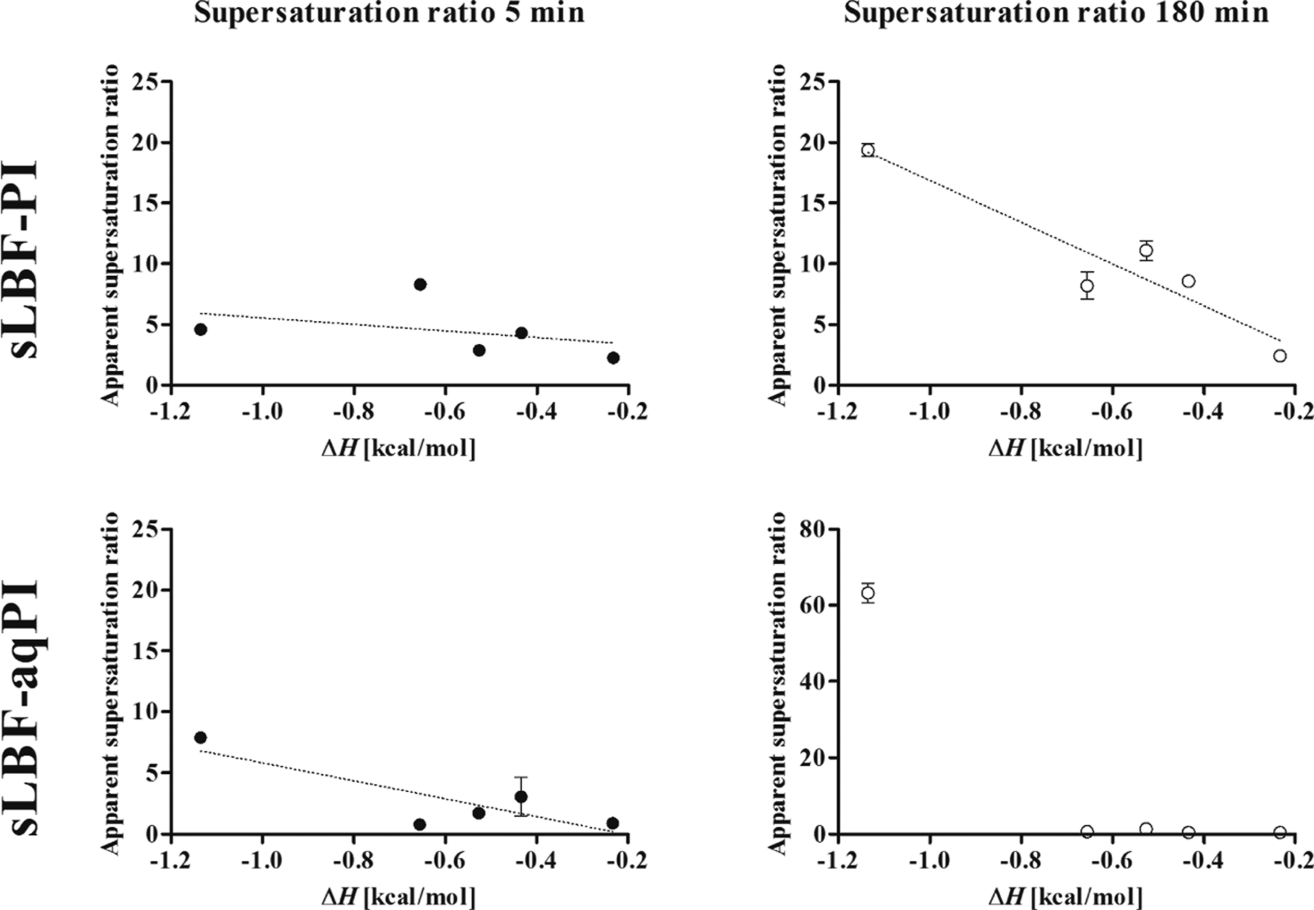
Relationship between excess enthalpy of mixing (calculated *in silico* using COSMOquick) and *in vitro* determined apparent supersaturation ratio after 5 and 180 min, respectively, for sLBF-PI added to FaSSIF (FaSSIF-PI) and sLBF added to FaSSIF-PI (sLBF-aqPI). Eudragit EPO was excluded from the data set due to the inability to generate supersaturation. Data are presented as mean ± SD, *n* = 3.

Based on the amount of venetoclax solubilized in the *in vitro* PI test, the general performance ranking was Pluronic F108 > PVP > HPMCAS ≥ PVP/VA > HPMC > Eudragit EPO in the case of sLBF-PI and Pluronic F108 > PVP/VA ≥ PVP ≥ HPMCAS ≥ HPMC > Eudragit EPO in the case of sLBF-aqPI. While this performance ranking was not in line with the *in silico* calculated COSMO-Rank, it should be acknowledged that Eudragit EPO was not able to generate supersaturated venetoclax concentrations in the presence of the lipid excipient. Therefore, this particular PI may not be suitable to enhance the sLBF performance. Subsequently, the relationship between the *in vitro* determined solubilized venetoclax and the *in silico* calculated COSMO-Rank was reassessed without Eudragit EPO. The results for sLBF-PI and sLBF-aqPI are summarized in Figure 4. It was evident that there is a trend that a higher COSMO-Rank, that is, higher interaction between venetoclax and the PIs resulted in a higher supersaturation ratio in the aqueous phase of the *in vitro* test in the case of sLBF-PI. From the *in silico* screen and *in vitro* experiments, a relationship between COSMO-Rank and venetoclax solubilization and supersaturation was less evident for sLBF-aqPI (no relationship was observed after 180 min). The results of both approaches (*in silico* and *in vitro*) indicated that for this lipid system an overly strong interaction between the drug and the PI may be less favorable, and in the case of Eudragit EPO, it resulted in the depletion of aqueous venetoclax concentrations.

### Impact of PIs on *In Vivo* Bioavailability of Venetoclax from sLBFs

**Figure 5:**
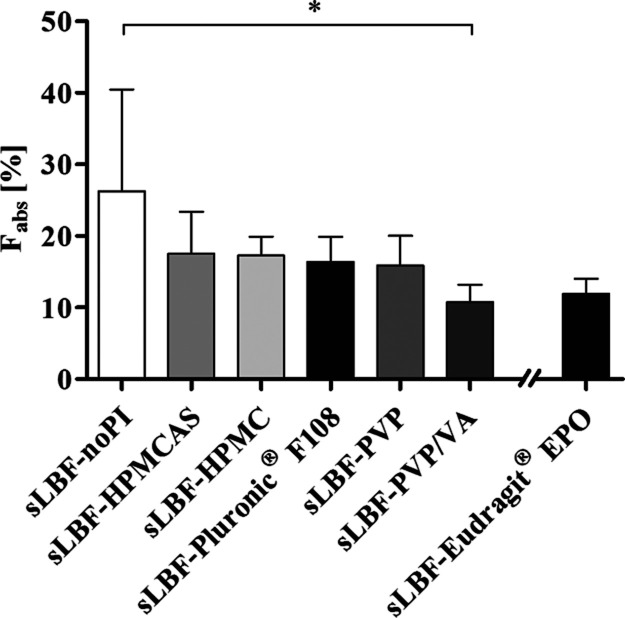
Absolute bioavailability (*F*_abs_) in landrace pigs for 100 mg venetoclax as a 6-way crossover with sLBF-noPI, sLBF-HPMC, sLBF-HPMCAS, sLBF-PVP, sLBF-PVP/VA, and sLBF-Pluronic F108 and an additional study including sLBF-Eudragit EPO. All data are presented as mean ± SD, where *n* = 5 (except sLBF-Eudragit EPO, where *n* = 3).

The aim of the *in vivo* study was to explore whether the *in vitro* observations would translate to *in vivo* using sLBF-PI across six different PIs. Venetoclax formulations (50 mg/mL) were prepared as a supersaturated Peceol solution alone (sLBF-noPI) or in combination with HPMC, HPMCAS, PVP, PVP/VA, Pluronic F108, and Eudragit EPO at a ratio of 1:1, respectively. PVP, PVP/VA, Pluronic F108, and Eudragit EPO were readily soluble in the sLBF, whereas HPMC and HPMCAS were not soluble in the sLBF and were therefore mixed in the sLBF prior to oral administration. An oral dose of 100 mg of venetoclax was used. Absolute bioavailability was determined with reference to the intravenous data that has been previously reported for landrace pigs.^46^ The absolute bioavailability as a function of each formulation is shown in Figure 5 and the associated pharmacokinetic parameters presented in Table 3. Individual plasma profiles for all tested formulations are shown in Figures S2 and S3 in the Supporting Information. While it was only feasible to conduct a full six by six way cross-over study in this experiment facility, a seventh study leg using Eudragit EPO was performed in a separate group of three pigs. In this case only mean comparisons were possible, whereas for all other groups formulation differences within each pig were performed.

**Table 3:** Pharmacokinetic Parameters for Venetoclax after Oral administration of 100 mg/pig to Male Landrace Pigs^a^

	pharmacokinetic parameters						
	sLBF-noPI	sLBF-HPMC	sLBF-HPMCAS	sLBF-PVP	sLBF-PVP/VA	sLBF-Pluronic F108	sLBF-Eudragit EPO^c^
*c*_max_ [μg/mL]	1.38 ± 0.84	0.92 ± 0.24	0.83 ± 0.20	0.81 ± 0.19	0.48 ± 0.67	0.80 ± 0.20	0.51 ± 0.28
*t*_max_ [h] (range)	6 (2–10)	6 (6–8)	8 (7–10)	8 (6–8)	9 (8–10)	9 (6–10)	10 (7–10)
AUC 0 h—inf. [μg·h/mL]	11.40 ± 6.15	7.39 ± 1.40	7.69 ± 2.53	6.90 ± 1.83	4.73 ± 1.08	7.19 ± 1.50	5.23 ± 1.60
MRT [h] (range)	8.64 (7.41–14.20)	10.17 (9.43–15.23)	10.96 (9.69–12.03)	10.44 (9.49–11.71)	11.48 (8.67–11.73)	10.62 (8.54–12.58)	13.67 (10.82–15.62)
MAT [h] (range)	4.76 (3.53–10.32)	6.29 (5.55–11.35)	7.08 (5.81–8.15)	6.56 (5.62–7.83)	7.61 (4.79–7.86)	6.74 (4.66–8.70)	9.79 (6.94–11.74)
*F*_rel_ [%]^b^	100	76.62 ± 32.34	78.86 ± 37.81	72.22 ± 30.56	52.95 ± 29.23	80.24 ± 42.16	68.87 ± 21.01^d^
*F*_abs_ [%]	26.28 ± 14.20	17.25 ± 2.63	17.57 ± 5.80	15.84 ± 4.18	10.76 ± 2.44	16.39 ± 3.47	11.95 ± 3.64
venetoclax appearance in plasma [h]	0.5 (0.5–2.0)	1 (0.5–1.5)	2 (1.5–2.0)	1.5 (0.5–2.0)	2 (2.0–3.0)	1.5 (0.5–3.0)	1.5 (0.5–3.0)

a Venetoclax was administered in a six-way crossover as a supersaturated Peceol solution (sLBF-noPI) and as sLBF with HPMC (sLBF-HPMC), HPMCAS (sLBF-HPMCAS), PVP (sLBF-PVP), PVP/VA (sLBF-PVP/VA), and Pluronic F108 (sLBF-Pluronic F108) and in a two-way crossover including Eudragit EPO (sLBF-Eudragit EPO). *T*_max_, MAT, MRT, and the appearance of venetoclax in the plasma are given as median (range), while all other parameters as mean ± SD (*n* = 5, except sLBF-Eudragit EPO, where *n* = 3).

b Relative to sLBF.

c sLBF-Eudragit EPO originated from a second *in vivo* study. sLBF for the second *in vivo* study has previously been published.^13^

d Relative to a previously published sLBF.^13^

The sLBF-noPI showed the highest mean bioavailability (26.3 ± 14.2%) compared to all other tested lipid formulations. An increased bioavailability was not observed by the incorporation of PIs but rather a trend toward a decreased oral bioavailability, albeit only the sLBF-PVP/VA formulation displayed statistically significant lower bioavailability relative to the sLBF-noPI (*p* < 0.05). The rank order of the oral bioavailability was sLBF-noPI ≥ sLBF-HPMCAS ≥ sLBF-HPMC ≥ sLBF-Pluronic F108 ≥ aLBF-PVP ≥ sLBF-Eudragit EPO ≥ sLBF-PVP/VA. The observed bioavailability for sLBF-noPI is in agreement with previously reported venetoclax bioavailability in fasted pigs (17.3 ± 5.5%). The time to reach the maximum plasma concentrations (*t*_max_), the mean residence time (MRT), and mean absorption time (MAT) tended to increase with lower oral bioavailability. In addition, a delayed appearance of venetoclax in the plasma was observed in the case of sLBF-PI. However, both observations were not statistically significant different. Overall, the results showed that the use of PIs did not enhance the sLBF formulation performance *in vivo*.

### sLBF Viscosity and Droplet Size Impact on *In Vitro* and *In Vivo* Performances

The *in vitro* and *in vivo* tested sLBF containing venetoclax and the evaluated PIs (sLBF-PI) were further assessed for their viscosity. In addition, upon dispersion in the FaSSIF, droplet size as an indicator for the available surface area and zeta potential as an indicator for the stability of the dispersion were investigated to probe the impact of these formulation parameters on the observed *in vivo* and *in vitro* behavior. The *in vitro* dispersion was performed as described in the *in vitro* testing for PIs by dispersing sLBF-PI in FaSSIF (1:50 dilution). PVP, PVP/VA, Pluronic F108, and Eudragit EPO were dissolved in sLBF, and HPMC and HPMCAS were ad hoc suspended in the sLBF due to a low solubility in the formulation. The results of the viscosity measurements are shown in Table 4, and the results of the droplet size and zeta potential measurements are shown in Table 5.

**Table 4:** Viscosity Measurements of Venetoclax Containing sLBF-PI Formulations at Shear Rates of 30, 150, and 300 1/s at 37 °C (Mean ± SD, *n* = 3)

	viscosity [mPa s]		
	30 1/s	150 1/s	300 1/s
sLBF-Eudragit EPO	326 ± 17	325 ± 16	324 ± 13
sLBF-HPMC	231± 64	130 ± 11	75 ± 3
sLBF-HPMCAS	139 ± 6	127 ± 6	122 ± 6
sLBF-PVP	498 ± 12	494 ± 12	493 ± 1
sLBF-PVP/VA	377 ± 9	372 ± 9	361 ± 6
sLBF-Pluronic F108	2439 ± 402	709 ± 34	312 ± 11
sLBF-noPI	93 ± 15	91 ± 15	89 ± 10

**Table 5:** Droplet Size and Zeta Potential of Venetoclax Containing sLBF-PI Dispersions in FaSSIF after 5 and 60 min of Dispersion^a^

	droplet size [nm]		PDI		zeta potential [mV]	
	5 min	60 min	5 min	60 min	5 min	60 min
sLBF-Eudragit EPO	107.2 ± 12.8	103.8 ± 3.6	0.53 (0.41–0.71)	0.77 (0.62–0.80)	–23.7 ± 1.2	–21.7 ± 1.0
sLBF-HPMC	175.7 ± 33.7	467.8 ± 132.6	0.30 (0.23–0.37)	0.35 (0.30–0.62)	–23.5 ± 3.0	–20.1 ± 1.9
sLBF-HPMCAS	167.8 ± 30.3	515.3 ± 150.3	0.44 (0.31–0.81)	0.47 (0.35–0.64)	–24.6 ± 1.3	–16.8 ± 1.2
sLBF-PVP	403.1 ± 156.8	200.3 ± 9.5	0.32 (0.25–0.61)	0.25 (0.16–0.32)	–14.1 ± 1.5	–16.2 ± 0.8
sLBF-PVP/VA	392.0 ± 35.0	256.1 ± 59.6	0.39 (0.30–0.45)	0.30 (0.21–0.32)	–24.3 ± 0.7	–21.2 ± 2.7
sLBF-Pluronic F108	325.0 ± 44.1	163.6 ± 19.8	0.35 (0.27–0.42)	0.31 (0.26–0.45)	–1.1 ± 0.2	–0.9 ± 0.2
sLBF-noPI	320.1 ± 48.6	197.0 ± 56.7	0.35 (0.31–0.42)	0.26 (0.21–0.30)	–31.2 ± 1.2	–33.9 ± 0.9

a PDI is given as median (range), while all other parameters as mean ± SD (*n* = 3).

At low (30 1/s) and medium (150 1/s) shear rates, the lowest viscosity was observed for the sLBF-noPI, while the highest viscosity was observed for sLBF-Pluronic F108. At a high shear (300 1/s), the lowest viscosity was observed for sLBF-HPMC (75 ± 3 mPa s) and the highest for sLBF-PVP (493 ± 1 mPa s). Thus, while a shear thinning effect was especially pronounced for sLBF-Pluronic F108 and sLBF-HPMC, in the case of Eudragit EPO, HPMCAS, PVP, PVP/VA, and sLBF-noPI, no significant viscosity decrease was observed with the increasing shear rate.

The droplet size formed on the dispersion in biorelevant media varied between formulations and was influenced by the dispersion time. After 5 min of dispersion, the highest droplet size was observed in the case of sLBF-PVP, while after 60 min of dispersion, the highest droplet size was observed for sLBF-HPMCAS. In the case of sLBF-noPI, an initial droplet size of 320 ± 49 nm was observed, which decreased to 197 ± 58 nm after 60 min of dispersion. In the case of the highly viscous formulations containing Pluronic F108, PVP, and PVP/VA, a high initial (after 5 min) droplet size was reached. However, the droplet size decreased on sampling after 60 min dispersion. In contrast, initially the low viscous sLBF-HPMC and sLBF-HPMCAS dispersed well with droplet sizes of approximately 170 nm, but a significant increase in the droplet size was observed after 60 min of dispersion. This indicated that viscosity influenced dispersibility of the formulations among other factors. (e.g., surfactant-like properties of Pluronic F108). In the case of sLBF-EPO, the droplet size appeared to be consistent over the 60 min; however, sampling for sLBF-EPO is not homogeneous due to large agglomerates, as illustrated in Figure S4.

Upon the dispersion of the sLBF-noPI and sLBF-PI, a negative zeta potential was obtained in all cases. The zeta potential was below −30 mV in the case of sLBF-noPI and between −30 and −20 mV in the case of sLBF with PVP/VA, HPMC, HPMCAS and Eudragit EPO. A higher zeta potential was observed in the case of sLBF-PVP (−14 ± 2 mV) and sLBF-Pluronic F108 (−1 ± 0.2 mV). Thus, on average no trends were observed on the zeta potential between formulations.

## Discussion

Bioenabling formulations that enhance the extent of oral drug absorption are increasingly required to meet the challenge of poor water-soluble properties of drugs emerging from discovery pipelines. Drug candidates which fail to meet Lipinski’s rule-of-five criteria are a common target for such bioenabling formulation strategies^2^ due to poor biopharmaceutical properties, resulting from low aqueous solubility and/or poor permeability. Ideally, a formulation design that rapidly generates elevated drug concentrations in the gastrointestinal fluids, that is, that generates the so-called “spring effect” to increase the concentration above the saturation solubility in gastrointestinal fluids and therein promote absorption.^27,29,30^ In recent years, LBF approaches have been mechanistically described as “supersaturable” drug delivery systems, on the basis of generating supersaturation following dispersion/digestion in the intraluminal environment. Indeed, it is widely recognized that dispersion/digestion can present a key risk to prolonged supersaturation, where there is a lower solubilization capacity of the post digestive milieu. In particular, LBFs that contain high % of co-solvents (such as LFCS type IV systems) are considered to be at the greatest risk of drug precipitation in the gastrointestinal tract. In order to prolong the onset of precipitation, PIs have been used.

The present study, therefore, aimed to explore the utility of PIs to enhance performance for sLBFs, where the API concentration exceeds thermodynamic solubility in the formulation. Our group and others have previously shown that sLBFs enhance oral bioavailability of poorly water-soluble drugs; albeit, there is a perceived risk of drug precipitation due to the systems being considered merely kinetically stable. To address this, in the current study, the utility of several PIs to enhance formulation performance of a sLBF of venetoclax has been evaluated *in vitro* and *in vivo*. We further calculated the excess enthalpy of mixing of the drug and various polymers to approximate the interaction between venetoclax and the PIs in a more complex aqueous environment. This approach facilitated a rationalization of *in vitro* testing, which was based on a solvent shift to assess the precipitation inhibitory effect.

The *in vitro* lipolysis confirmed the ability of sLBF to generate supersaturated aqueous phase concentrations of venetoclax during dispersion and digestion, which exceeded the experimentally determined apparent solubility in FaSSIF (Figure 1) and the reported values for the experimentally determined amorphous solubility in FaSSIF (20.7 ± 0.5 μg/mL, pH 5.3; 33.7 ± 13.5 μg/mL, pH 6.9) and FeSSIF (26.4 ± 0.2 μg/mL, pH 5.3; 54.6 ± 2.0 μg/mL, pH 6.9).^44^ Such elevated concentrations were not observed for an aqueous and lipid-based suspension and therefore confirm the ability of sLBF to generate supersaturated drug concentrations on dispersion/digestion in intestinal fluids. Additionally, as a class III glass former^13^ and due to the high molecular weight, venetoclax tends to crystallize more slowly. Moreover, venetoclax has been reported to undergo liquid–liquid-phase separation at concentrations above the amorphous solubility, and it was assumed that supersaturation is maintained for a duration that is physiologically promising (24 h).^44^ While in the case of the sLBF, the physical state of the drug in such a separated drug-rich phase is unknown, it can potentially serve as a reservoir of the absorbable drug.^44^ The co-existence of a drug-rich phase and an aqueous phase at amorphous solubility (which were not separated) may explain the amount of venetoclax measured above the amorphous solubility in this test setup.

The *in vitro* PI screening method revealed that the incorporation of the PI into the sLBF resulted in a higher venetoclax concentration in the aqueous phase compared to the addition of sLBF to the media containing pre-dissolved PI. Interestingly, the sLBF formulation demonstrated an initial supersaturated venetoclax concentration, which was maintained for up to 2 h even in the absence of a PI (i.e. sLBF-noPI). These findings are in line with the observations during *in vitro* lipolysis and confirm the ability of sLBF approaches to generate supersaturation, that is, to act as a spring. The incorporation into the sLBF (sLBF-PI) of the PIs: PVP/VA, HPMCAS, PVP, Pluronic F108, and HPMC, proved beneficial for venetoclax, resulting in prolonged supersaturation *in vitro* (apparent supersaturation ratio >2.4 for all polymers after 180 min of dispersion). However, the incorporation of Eudragit EPO resulted in a decreased venetoclax concentration in the aqueous phase to below FaSSIF solubility (0.08-fold reduction relative to FaSSIF solubility). It was also noticeable that the sLBF-Eudragit EPO was poorly dispersible in FaSSIF, forming a two-phase system with drug-rich agglomerates dispersed in buffer (Figure S4). In contrast, all the sLBF-noPI and sLBFs containing PIs dispersed consistently in FaSSIF to form a homogenous dispersion on mixing. One possible explanation for the significantly lower venetoclax concentrations observed for the sLBF-Eudragit EPO system may reflect the poor dispersion in FaSSIF. This observation suggested that venetoclax may have remained within the lipid-rich agglomerates and was not released from the formulation into the aqueous phase. Additionally, Eudragit EPO (in the unionized form, expected in neutral and alkaline conditions, p*K*_a_ ∼ 6^47^) and venetoclax are relatively lipophilic and interact strongly with each other, as indicated by the calculated negative excess enthalpy of the interaction with the *in silico* tool, which may have further promoted the drug retention in the lipid phase. Another aspect is that the unionized Eudragit EPO was not expected to swell in aqueous medium and such a swelling of a more hydrophilic polymer is likely to contribute to the performance of a PI. This complexity in aqueous medium was not captured by the simple mixing enthalpy calculations of binary drug-polymer systems.

The *in vivo* study demonstrated that the highest mean oral bioavailability was obtained with the sLBF-noPI. However, a high variability for *c*_max_, *t*_max_, and AUC was observed. The sLBF-PI formulations showed a trend toward a decreased oral bioavailability, when compared to sLBF-noPI. The overall bioavailability of 26.3 ± 14.2% for sLBF-noPI is in line with previous reports of venetoclax bioavailability in large animal models.^13,48^ However, the results of the present study were unexpected because previously published studies exploring the inclusion of PIs with LBF resulted in an increased bioavailability.^23−25^ However, in the previously reported studies, LFCS-type IIIB/IV LBF systems were used, which contained high amounts of co-solvents and exhibited a high risk of precipitation due to the dilution effect upon dispersion.^26^ Therefore, this confirmed that the ability of an oil-only sLBF to generate supersaturated concentrations of venetoclax *in vitro* was translated to increased absorption *in vivo* and that the duration of the supersaturation for the sLBF was sufficient to obviate the inclusion of a PI. This observation that a PI was not required may be specific for venetoclax, given that the drug as a class III glass former has a low tendency to crystallize.^44^ Hence, for other drugs such as poor glass formers as well as sLBF containing higher proportions of co-solvents further studies are required to assess whether sLBF approaches with PIs are needed to maximize absorption and mitigate a perceived risk of precipitation *in vivo*.

The median MAT for the sLBF-PI formulations was higher among all PI containing formulations relative to sLBF-noPI (Table 3). Overall, given the variability in the absorption rate of venetoclax in each group, no statistically significant differences were observed. However, it would appear that the inclusion of a PI may present a risk of a delayed absorption that may reflect a delay of drug release from the formulation (as evident by the delayed onset of venetoclax appearance in the plasma for sLBF-PI, Table 3). An explanation for this observation might be a combined effect of the extent of the drug–polymer interaction and a reduced polymer swelling. The incorporation of the PI into the sLBF may have reduced polymer swelling (which normally happened in the aqueous media) and caused trapping of the drug. In addition, the interaction between the PIs and the drug reduces the diffusion of both PI and the drug, and hence, the partitioning of venetoclax and PIs from the sLBF to the aqueous phase.

The partitioning may be further reduced due to an increased viscosity of the sLBF-PI formulations as observed in this study (Table 4), which further decreases the diffusion of the drug from the inner part of the lipid droplets toward the bulk. The viscosity data revealed that at low and moderate shear, which would be expected *in vivo* in pigs,^49^ the viscosity for all tested sLBF-PI was higher compared to LBF-noPI. In addition, a higher viscosity might also lead to a decreased dispersibility (e.g., as observed *in vitro* in the case of Eudragit EPO, Figure S4, or PVP and Pluronic F108, Figure 3), and subsequently digestibility and drug release. While a higher viscosity of sLBF-PVP, sLBF-PVP/VA, and sLBF-Pluronic F108 and a lower viscosity of sLBF-HPMC and sLBF-HPMCAS resulted in an initial high- and low-lipid droplet size upon dispersion, respectively, the results of the lipid droplet size analysis are not apparent in the case of sLBF-noPI and sLBF-Eudragit EPO. In addition, the observed change of the droplet size during a prolonged period of dispersion in this study indicated that besides the initial dispersibility and viscosity other factors such as the complex interactions between PI, lipid, drug, and the dispersion media might have an impact on the dispersion and drug release of the sLBF-PI. However, further studies are needed to explore the effect of these parameters on sLBF performance *in vivo*.

From the *in vivo* data, it appears that the solubility of the polymer in the sLBF may have impacted the bioavailability. In the cases of PIs being soluble in the sLBF, that is, Pluronic F108, PVP, PVP/VA, and Eudragit EPO dissolved completely in the sLBF, a lower bioavailability was observed when compared to the PIs that showed a lower solubility in the sLBF, that is, HPMC and HPMCAS formed suspensions in the sLBF. One explanation for this observation may be that a PI that is soluble in the lipid vehicle can, in combination with a high drug affinity of the PI, lead to a drug retention in the vehicle instead of showing a more favorable PI functionality of reducing precipitation once the sLBF has dispersed in the intraluminal fluids. On the other hand, a more hydrophilic polymer that is suspended in the sLBF may lead to polymer swelling upon aqueous dispersion to allow for drug release and the intended PI functionality. Further studies exploring polymer solubility in sLBF and its effect on drug release may therefore be merited to fully predict the impact of PIs on *in vivo* performance.

Overall, a relationship between the *in silico* calculated excess enthalpy of mixing and the *in vitro* determined supersaturation ratios and amounts of venetoclax solubilized was established in the case of PIs that generated supersaturated concentrations. This study showed that with increasing “COSMO-Rank” (i.e., lower excess enthalpy of mixing), higher apparent supersaturation ratios and higher amounts of solubilized venetoclax were obtained. Because Eudragit EPO resulted in undersaturated aqueous solutions (venetoclax concentration below FaSSIF saturation solubility) due to a lower release from the formulation, the PI was not considered in the analysis of the relationship between *in silico* and *in vitro*. However, the result of Eudragit EPO showed a simple consideration of the high negative excess enthalpy for a more lipophilic polymer in sLBF and should be interpreted with care regarding PI performance.

The deviation of the *in vivo* results from the *in silico* calculations by COSMOquick software may reflect an oversimplification of the calculated parameters. The calculations considered the interactions between the drug and the PI, but the interaction between the drug or the PI with formulation excipients, water, or the components of gastrointestinal fluids such as bile salts and phospholipids were not taken into account. While this might have not been crucial for an *in vitro* dispersion experiment, the digestion of the lipid excipients *in vivo* further increased the complexity of the gastrointestinal fluids, by releasing fatty acids and other digestion products in the gastrointestinal environment. It is, hence, unclear whether a drastic simplification to solely the drug and PI by the selected COSMOquick approach is applicable for LBFs to define PIs. Nevertheless, the current *in silico* approach may be useful for type IV LBFs/sLBFs that contain less digestible excipients, that is, co-solvents, and may be a quick screening tool to reduce the initial PI choice to a reasonable number, which subsequently can be tested *in vitro* and *in vivo*. Furthermore, the complementing *in vitro* and *in silico* techniques used in the present study may be helpful in understanding the formulation behavior of sLBFs *in vivo*.

A lack of PI impact on the increasing bioavailability may also reflect that the *in vitro* model was poorly predictive of the *in vivo* situation. The reasons for the *in vitro* test not being predictive of a reduced overall drug absorption in the presence of PIs can be manifold such as (a) more complex intestinal conditions *in vivo*,^50^ (b) a lack of the absorptive sink *in vitro*,^51^ or (c) a venetoclax specific effect. The employed *in vitro* test exhibited a high drug and lipid load as well as higher hydrodynamics compared to *in vivo*. Furthermore, especially, the high venetoclax concentration in the presence of Pluronic F108 may have been influenced by the surfactant properties of the polymer leading to an overpredictive result *in vitro*. In addition, a solvent shift may meet the industry need for a fast screening tool; however, in the case of LBFs, it is not physiologically relevant. A combined dispersion/digestion setup^26^ or a dispersion/digestion setup with an absorptive sink^52−54^ may provide more mechanistic, albeit lower throughput, results. A further limitation of the study may have been the incorporation of the PI into the sLBF. While this decision was guided by *in vitro* data, in light of the *in vivo* results, it is unclear whether separating the PI and sLBF may have conferred advantages *in vivo*.

## Conclusions

The formulation approach of using PIs to prolong supersaturation is well recognized for amorphous formulations but less well explored for sLBFs. The present study was applied *in silico*, *in vitro*, and *in vivo* models to extend the concept of PIs in an oil-only sLBF. An *in silico* tool was used for an initial PI selection and aided in explaining the low free venetoclax concentration *in vitro* in the case of the sLBF with Eudragit EPO. It was found that the strong predicted interaction between the drug and the polymer may have led to an overall reduction in the venetoclax release from the formulation. In addition, the *in vitro* PI screening tool showed that the incorporation of the PI into the sLBF yielded higher free drug concentrations compared to the separate addition of the PI. While the *in vitro* screening showed prolonged supersaturated venetoclax concentrations in the presence of PIs in five out of six cases, an *in vivo* trend toward a lower overall bioavailability was observed for PI containing formulations, indicating that incorporating a PI into the sLBF was not necessary. The oral bioavailability of venetoclax was the highest for the PI-free sLBF-noPI, which suggests that the reduced oral absorption due to precipitation from an oil-only sLBF was low.

## Supporting Information Available

*In vitro* lipolysis of venetoclax; individual plasma concentration time profiles; PI test samples of sLBF-PI with HPMC, Eudragit EPO, and HPMCAS; and thermal properties of venetoclax (PDF)

## Abbreviations

COSMO: conductor-like screening model
LBF: lipid-based formulation
PI: precipitation inhibitor
sLBF: supersaturated lipid-based formulation
sLBF-PI: supersaturated lipid-based formulation with an incorporated precipitation inhibitor
sLBF-aqPI: supersaturated lipid-based formulation with a pre-dissolved precipitation inhibitor in dispersion media
