# Supplementary material for: In Silico, In Vitro, and In Vivo Evaluation of Precipitation Inhibitors in Supersaturated Lipid-Based Formulations of Venetoclax
Source: Mol Pharm. 2021 Apr 23;18(6):2174–88. doi: 10.1021/acs.molpharmaceut.0c00645 (PMC8289286; doi:10.1021/acs.molpharmaceut.0c00645)
Supplement: Supplementary file 1 — mp0c00645_si_001.pdf [file mp0c00645_si_001.pdf]

**Supporting information**

# *In silico*, *in vitro* and *in vivo* evaluation of precipitation inhibitors in supersaturated lipid-based formulations of venetoclax

*Niklas J. Koehl*[1,2], *Laura J. Henze*[1,3], *Harriet Bennett-Lenane*[1], *Waleed Faisal*[1,4], *Daniel J. Price*[5,6], *René Holm*[2,7,8], *Martin Kuentz*[9], *Brendan T. Griffin*[1,*]

[1] School of Pharmacy, University College Cork; College Road, Cork, T12 YN60, Ireland

[2] Drug Product Development, Janssen Research and Development, Johnson & Johnson, Turnhoutseweg 30, 2340 Beerse, Belgium

[3] Analytical Development, Janssen Research and Development, Johnson & Johnson, Turnhoutseweg 30, 2340 Beerse, Belgium

[4] Faculty of Pharmacy, Minia University, Minia, Egypt

[5] Merck KGaA, Frankfurter Str. 250, 64293 Darmstadt, Germany

[6] Institution of Pharmaceutical Technology, Goethe University Frankfurt, Max-von-Laue-Strasse 9, 60439 Frankfurt am Main, Germany

[7] Department of Science and Environment, Roskilde University, 4000 Roskilde, Denmark

[8] Department of Physics, Chemistry and Pharmacy, University of Southern Denmark, Campusvej 55, 5230 Odense, Denmark

[9] University of Applied Sciences and Arts Northwestern Switzerland, Institute of Pharma Technology, Hofackerstrasse 30, 4132 Muttenz, Switzerland

*Corresponding author: Brendan Griffin, University College Cork, School of Pharmacy, Cavanagh Building, College Road, Cork, T12 YN60, Ireland

Phone: +353 (0) 21 490 1657, Fax: +353 (0) 21 490 1656, e-mail: Brendan.Griffin@ucc.ie

| Content | Page |
|---|---|
| *In vitro* lipolysis: Venetoclax concentrations in the different phases (Figure S 1) | 2 |
| Individual plasma concentration time profiles, study 1 (Figure S 2) | 3 |
| Individual plasma concentration time profiles, study 2 (Figure S 3) | 3 |
| PI test samples of sLBF-PI with HPMC, Eudragit® EPO and HPMCAS (Figure S 4) | 4 |
| Thermal properties of venetoclax (Table S 1) | 4 |

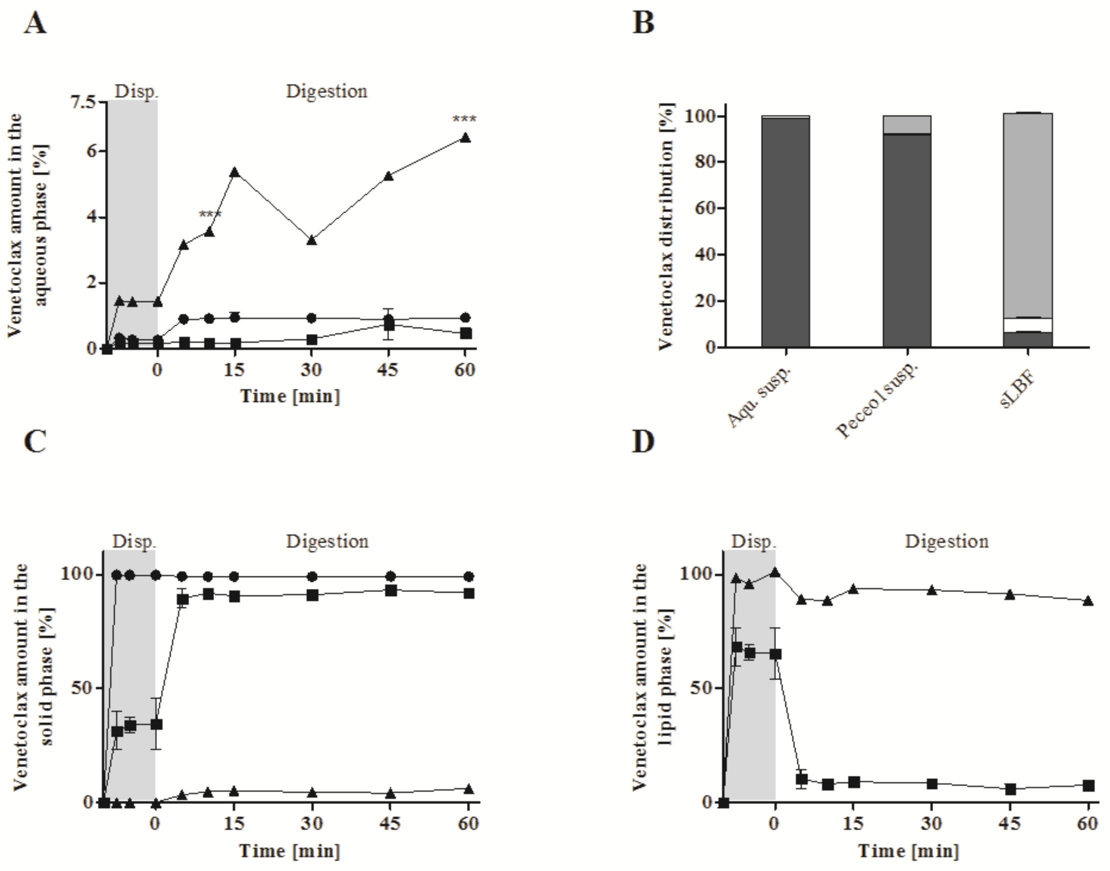


**Figure S 1.** *In vitro* lipolysis of venetoclax formulated as supersaturated Peceol® solution (sLBF) (▲), as well as previously reported aqueous suspension (●), Peceol® suspension (■).[13] A: % of venetoclax in the aqueous phase, B: Distribution of venetoclax into the different phases after 60 min of lipolysis (black: solid phase, white: aqueous phase, grey: lipid phase), C: % of venetoclax in the solid phase, D: % of the venetoclax in the lipid phase. All experiments were run with n = 3 and results are shown as mean ± SD.

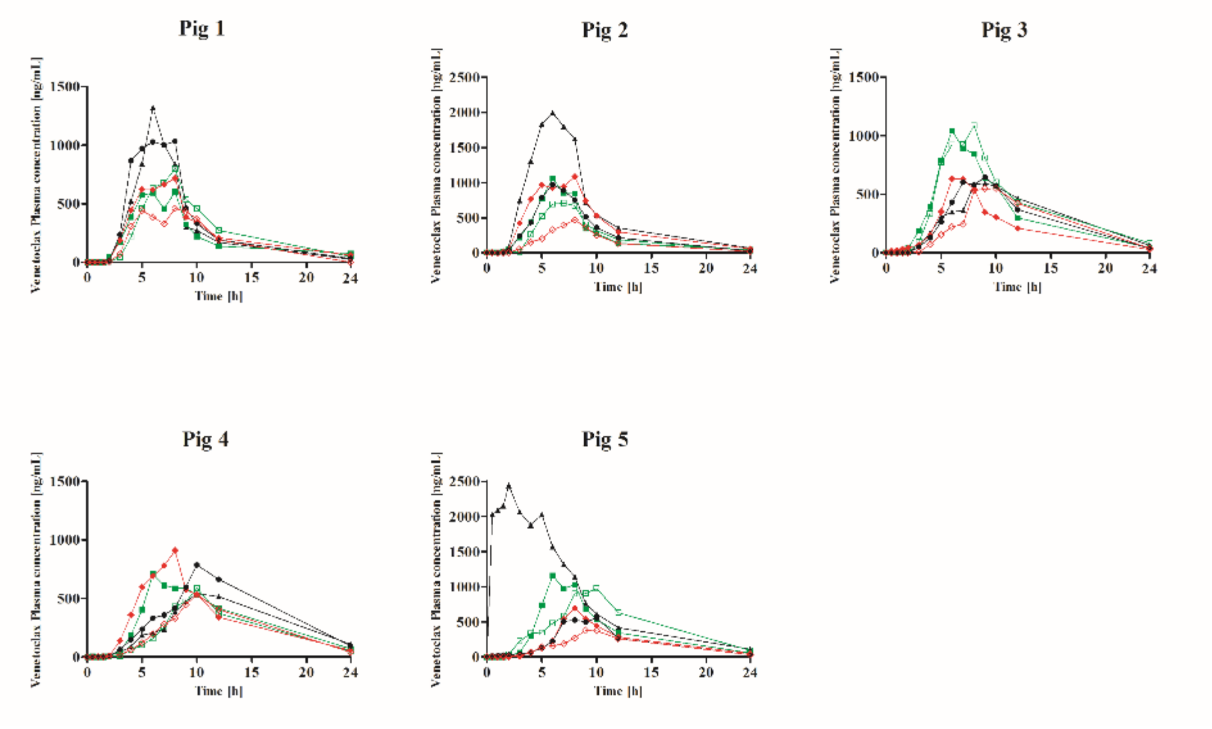


**Figure S 2.** Individual plasma concentration profile from 0 – 24 h of 100 mg venetoclax in landrace pigs for the tested formulations. sLBF-noPI (▲), sLBF-HPMC (■ - green), sLBF-HPMCAS (□ - green), sLBF-PVP (♦ - red), sLBF-PVP/VA (◊ - red), sLBF-Pluronic® F108 (●).

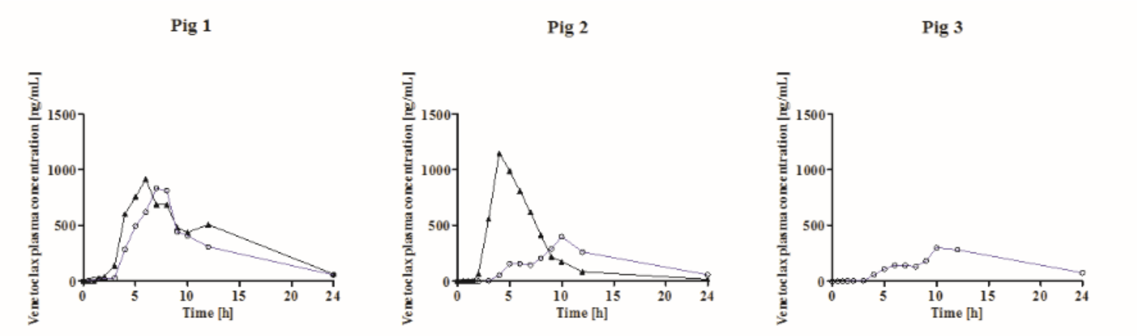


**Figure S 3.** Individual plasma concentration time profiles from 0 - 24 h of 100 mg venetoclax in landrace pigs for the tested formulations. sLBF-noPI (▲), sLBF-Eudragit® EPO (○ - blue). Due to an administration error, the sLBF-noPI of pig 3 was not obtained. sLBF-noPI has previously been reported. [13]

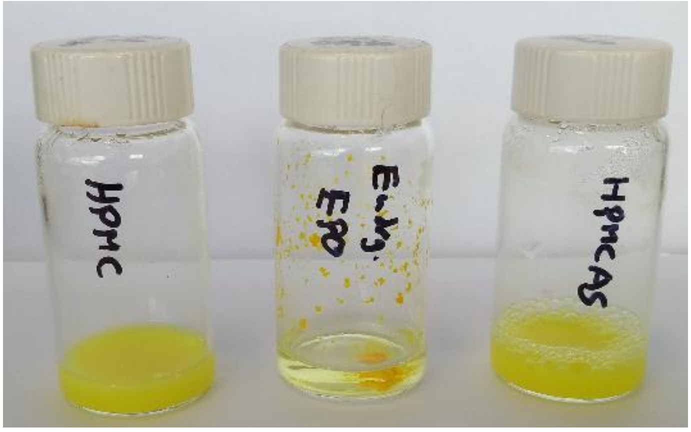


**Figure S 4.** PI test samples of sLBF-PI with HPMC, Eudragit® EPO and HPMCAS after 180 min in FaSSIF. Agglomeration of Eudragit® EPO can be observed.

**Table S 1.** Thermal properties of venetoclax. Melting point ($T_m$), Enthalpy of fusion ($\Delta H_{fus}$), Entropy of fusion ($\Delta S_{fus}$).

| | $T_m$ [ºC] | $\Delta H_{fus}$ [kJ/mol] | $\Delta S_{fus}$ x$10^{-2}$ [kJ/mol/K] |
|---|---|---|---|
| Venetoclax batch 1810004 | 139.1 ± 0.03 | 19.2 ± 0.03 | 4.7 ± 0.01 |

ACKNOWLEDGEMENTS

N.J. Koehl, L.J. Henze, D.J. Price, B.T. Griffin, R. Holm and M. Kuentz are part of the PEARRL European Training network, which has received funding from the Horizon 2020 Marie Sklodowska-Curie Innovative Training Networks programme under grant agreement No. 674909.
